# Tailoring ω-3 fatty acid enrichment through genipin, glutaraldehyde, and glyoxyl linked immobilization of *Rhizomucor miehei* lipase on MWCNTs

**DOI:** 10.1007/s11274-026-05011-y

**Published:** 2026-05-12

**Authors:** Deniz Yildirim, Ahmet Tülek, Nurettin Paçal, N. Ece Varan Faki, Ali Toprak, Dilek Alagöz, Ramazan Bilgin

**Affiliations:** 1 Faculty of Engineering, Department of Chemical Engineering, Cukurova University, Adana, 01330 Türkiye; 2 Faculty of Science and Letters, Department of Chemistry, Cukurova University, Adana, 01330 Türkiye; 3 Faculty of Engineering and Natural Sciences, Department of Molecular Biology and Genetics, Kütahya Health Sciences University, Merkez, Kütahya, 43100 Türkiye; 4 Department of Biology, Iğdır University, Postgraduate Education Institute, Iğdır, 76000 Türkiye; 5 Acigol Vocational School, Nevsehir Haci Bektas Veli University, Acigol, Nevsehir, 50140 Türkiye; 6 Imamoglu Vocational School, Cukurova University, Imamoglu, 01700 Adana, Türkiye

**Keywords:** *Rhizomucor miehei* lipase, Multi-walled carbon nanotubes, Fish oil, EPA and DHA

## Abstract

Efficient enrichment of eicosapentaenoic acid (EPA) and docosahexaenoic acid (DHA) from natural sources remains a major challenge for sustainable ω-3 fatty acid production. This study reports the immobilization of *Rhizomucor miehei* lipase (RML) on multi-walled carbon nanotubes (MWCNTs) using three distinct coupling chemistries, including genipin (MWCNT/Gen@RML), glutaraldehyde (MWCNT/Glu@RML), and glyoxyl (MWCNT/Gly@RML). The resulting nanostructured biocatalysts were systematically evaluated for the selective enrichment of docosahexaenoic acid (DHA) and eicosapentaenoic acid (EPA) from commercial fish oil. The immobilized RML derivatives were characterized using FTIR, SEM, SEM-EDS, and TGA analysis. The maximum immobilized protein amounts were approximately 8.4, 8.1, and 8.6 mg g⁻^1^ support for MWCNT/Gen@RML, MWCNT/Glu@RML, and MWCNT/Gly@RML, respectively, when 10 mg of protein was initially loaded per gram of support. The optimal pH was 7.5 for free RML and all immobilized RML derivatives, and the optimal temperatures were 45 °C for free RML, 55 °C for MWCNT/Glu@RML, and 60 °C for MWCNT/Gen@RML and MWCNT/Gly@RML. Thermal stability improved markedly for all immobilized derivatives, increasing by approximately 34.6, 25.5, and 44.4 fold for MWCNT/Gen@RML, MWCNT/Glu@RML, and MWCNT/Gly@RML, respectively at 60 °C. Kinetic analysis indicated that MWCNT/Glu@RML achieved the highest catalytic efficiency (*k*_*cat*_/*K*_*m*_) of 19.2 mM⁻^1^ min⁻^1^, while MWCNT/Gen@RML exhibited superior reusability, retaining 75% of its initial activity after five cycles. During fish oil hydrolysis, MWCNT/Gen@RML provided the fastest midterm accumulation (92.85% total EPA + DHA at 36 h), whereas Glu-RML delivered the highest endpoint yield (82.33% at 48 h). The immobilized systems also enabled selective enhancement of either DHA or EPA, depending on coupling agent type and enzyme loading. Overall, the use of functionalized MWCNTs as supports, combined with mild natural and synthetic coupling agents produced stable high-performing lipase biocatalysts, and offered a sustainable route for the concentration of ω-3 fatty acids under mild, environmentally friendly conditions.

**Supplementary Information:**

The online version contains supplementary material available at 10.1007/s11274-026-05011-y.

## Introduction

Fish oil has long been utilized as a dietary supplement owing to its well-documented health benefits. Among its principal components, the long-chain ω-3 polyunsaturated fatty acids (ω-3 LC-PUFAs)—namely eicosapentaenoic acid (20:5n-3, EPA) and docosahexaenoic acid (22:6n-3, DHA)—are recognized for their critical physiological and biochemical roles (Alijani et al. 2025). These include cardioprotective effects, enhancement of brain function and retinal development, anticancer and anti-inflammatory activities, and regulation of lipid metabolism. In addition to their involvement in diverse cellular processes, EPA and DHA are known to influence the expression and regulation of numerous key genes (Kosti et al. 2022; Kar et al. 2023). Although the regulatory impacts of ω-3 fatty acids on human health, particularly EPA and DHA, are well documented, humans cannot efficiently synthesize these long-chain fatty acids, as the conversion of α-linolenic acid (ALA) is limited and insufficient to meet physiological demands (Gonzalez-Soto and Mutch 2021; Qin et al. 2023). PUFAs are mainly derived from fish and algal oils; however, their proportion is relatively low (typically ≤ 30%), and these sources may also contain impurities that compromise stability (Swetha and Mathanghi 2024). With the growing demand from the nutraceutical, functional food, and pharmaceutical industries, the need for concentrated, high-purity ω-3 fatty acids has increased substantially. Consequently, the development of efficient methods to obtain concentrated EPA and DHA has become indispensable (Oliver et al. 2020).

Commercial EPA and DHA concentrates are generally produced in ethyl ester or acylglycerol forms, both of which exhibit high bioavailability in humans. To eliminate non-triglyceride components that impair stability, fish oils are subjected to physical and chemical refining processes. Nevertheless, these processes, particularly molecular distillation conducted at high temperatures and pressures, pose risks of oxidative degradation and isomerization of PUFAs, especially EPA and DHA (Yi et al. 2023; Alijani et al. 2025). Therefore, recent research has increasingly focused on alternative enrichment strategies that can preserve these sensitive compounds (Ongis et al. 2025). Enzymatic approaches represent a promising and sustainable option, offering high specificity under mild reaction conditions that minimize oxidation and unwanted by-products (Castejón and Señoráns 2020).

Lipases (EC 3.1.1.3) catalyze not only ester bond hydrolysis but also a range of reactions including esterification, transesterification, interesterification, and aminolysis (Stergiou et al. 2013; Baena et al. 2022; Tacias-Pascacio et al. 2025). Their broad substrate specificity and adaptability to diverse reaction environments have made them valuable in various industries, including food, cosmetics, biofuels, and pharmaceuticals (Xu et al. 2025; Abdelaziz et al. 2025). Recent studies have shown that certain lipases can selectively hydrolyze or transesterify long-chain ω-3 fatty acids such as EPA and DHA from fish oil, thereby enabling their enrichment (Yi et al. 2023; Karia et al. 2024; Yu et al. 2025). Through lipase mediated reactions, saturated and monounsaturated fatty acids can be selectively removed, leading to PUFA enrichment, while the formation of mono and diacylglycerols may further enhance bioavailability (Qiao et al. 2024; Yu et al. 2025; Yan et al. 2025).

Despite their advantages, large-scale applications of lipases are often constrained by issues such as limited activity yield, high production costs, and difficulties in recovery and reuse of free enzymes. To address these limitations, a variety of immobilization methods including covalent binding, adsorption, and entrapment, have been explored (Ismail and Baek 2020). When appropriate supports and binding chemistries are employed, immobilization enhances operational and thermal stability, facilitates enzyme-product separation, improves reusability across multiple cycles, and reduces overall process costs (Chandra et al. 2025). Lipases are widely immobilized on hydrophobic materials through physical adsorption, a process strongly associated with the phenomenon of interfacial activation. This structural rearrangement promotes efficient interaction with hydrophobic substrates and often results in enhanced catalytic activity. However, immobilization based solely on hydrophobic adsorption relies on relatively weak non-covalent interactions between the enzyme and the support surface. As a consequence, enzyme desorption may occur under operational conditions, particularly during prolonged reactions or repeated catalytic cycles. To overcome these limitations, covalent immobilization strategies have increasingly been explored, as they can provide stronger enzyme–support interactions and significantly improve enzyme stability, operational durability, and reusability in industrial biocatalytic systems (Schmid and Verger 1998; Verger 1997). Multi-walled carbon nanotubes (MWCNTs) have emerged as particularly attractive carriers due to their high surface-to-volume ratio, mechanical strength, dispersibility, chemical inertness, biocompatibility, conductivity, and minimal enzyme leakage (Rathinavel et al. 2021). In addition, their strong adsorption capacity and low mass-transfer resistance further enable high enzyme loading efficiency (Hughes et al. 2024).

Chemical cross-linkers and functional spacer arms play a central role in enzyme immobilization by improving enzyme orientation and stability. Their performance and suitability vary depending on the catalytic system and regulatory framework, which are critical considerations in food-related bioprocessing. Glutaraldehyde is one of the most widely used crosslinking agents for covalent immobilization of enzymes and proteins on amino-activated supports. Immobilization typically involves reaction between aldehyde groups and nucleophilic residues on the protein surface, primarily terminal amino groups and ε-amino groups of lysine residues (Migneault et al. 2004; Sheldon 2010; Barbosa et al. 2013; Sampaio et al. 2022). However, the immobilization on these supports may occur through a combination of ionic adsorption and covalent interactions, depending on the immobilization conditions and the structural characteristics of the support (Monsan 1978). Although glutaraldehyde-based immobilization is highly efficient, regulatory and safety concerns generally restrict its direct use in food processing unless it is shown that no free glutaraldehyde or toxic residues remain in the final product. Glyoxyl-activated supports represent another important strategy for covalent enzyme immobilization. In this approach, aldehyde groups generated on the support surface react with nucleophilic amino groups of the enzyme under alkaline conditions, typically leading to the formation of multiple covalent bonds between the protein and the carrier surface. This multipoint covalent attachment significantly enhances enzyme stabilization by restricting conformational flexibility and preventing structural unfolding during catalytic operation. (Guisán 1988; Mateo et al. 2006). Unlike glutaraldehyde, glyoxyl groups are anchored to the support itself, rather than being added as free cross-linkers. Glyoxyl supports are considered safe, as no free toxic cross-linker is added. Their strong stabilizing effects and clean chemical profiles make them widely used for immobilizing food enzymes, such as lipases. Genipin is a naturally occurring crosslinking agent derived from *Gardenia jasminoides* fruits and has attracted considerable attention as a low-toxicity alternative to traditional aldehyde-based crosslinkers. Genipin reacts with primary amine groups of proteins to form stable heterocyclic crosslinked structures through nucleophilic attack and subsequent polymerization reactions. Compared with glutaraldehyde, genipin exhibits significantly lower cytotoxicity while still providing strong and stable covalent linkages between enzymes and supports. This property makes genipin particularly attractive for applications in food processing, biotechnology, and biomedical fields where biocompatibility is important (Du et al. 2020; Ahmed et al. 2024; Wahba 2024).

The aim of this study was to develop and systematically evaluate a series of RML biocatalysts immobilized on chemically functionalized MWCNTs. RML was selected due to its widespread industrial application, well characterized catalytic properties, pronounced sn-1,3 regioselectivity toward triglycerides, and proven efficiency in PUFA enrichment processes, making it a suitable enzyme for the selective enrichment of EPA and DHA from commercial fish oil (Rodrigues and Fernandez-Lafuente 2010; Yousefi et al. 2020). RML was immobilized on MWCNTs using three distinct coupling chemistries, such as genipin, glutaraldehyde, and glyoxyl and the resulting biocatalysts were systematically characterized and compared in terms of physicochemical properties, catalytic efficiency, thermal stability, reusability, and selective enrichment of ω-3 fatty acids. Despite extensive research on enzymatic enrichment of ω-3 fatty acids from fish oil, comparative studies evaluating different coupling strategies on MWCNT-based supports for ω-3 enrichment are scarce. Notably, genipin-functionalized MWCNTs are introduced here for the first time as a low-toxicity and food-compatible immobilization platform for lipase-mediated ω-3 enrichment, providing new insights into how spacer arm chemistry can be used to tune the catalytic behaviour and product distribution of immobilized lipases. By establishing a direct link between immobilization chemistry and product selectivity, this study offers a tunable and scalable biocatalytic approach for the tailored enrichment of EPA and DHA under mild and sustainable processing conditions.

## Materials and methods

MWCNT-NH₂ and MWCNT-OH nanomaterials were purchased from Nanografi (Türkiye). Genipin (98%) was supplied by Pharmatech Co., Limited (USA). Lipase from *Rhizomucor miehei* (CAS number: 9001-62-1, Product number: L4277, ≥ 20,000 U/g), cis-4,7,10,13,16,19-docosahexaenoic acid, and cis-5,8,11,14,17-eicosapentaenoic acid were obtained from Sigma-Aldrich Chemie GmbH (Germany). BCA protein assay kit was supplied by Thermo Fisher Scientific (UK). All other chemicals were of analytical grade and purchased from Merck (Germany).

### Activation of immobilization supports

The activation of MWCNT-NH₂ with genipin was performed as described by Tülek et al. (2025). One gram of MWCNT-NH₂ was dispersed in 10 mL sodium phosphate solution (50 mM, pH 6.5) containing 1 mg/mL genipin and incubated at 60 °C for 2 h in a temperature-controlled orbital shaker with agitation at 100 rpm. Subsequently, the resulting support (MWCNT/Gen) was collected by vacuum filtration and rinsed with distilled water. The washing step was continued until no genipin was detected in the filtrate at 280 nm. The resulting MWCNT/Gen particles were then stored at room temperature until use.

Activation of MWCNT-NH₂ with glutaraldehyde was performed as described by Alagöz et al. (2021). One gram of MWCNT-NH₂ was treated with 25 mL sodium phosphate solution (50 mM, pH 7.0) containing 1.25% glutaraldehyde at 25 °C. The mixture was continuously stirred at 100 rpm using a magnetic stirrer for 2 h. Subsequently, the resulting support (MWCNT/Glu) was collected by vacuum filtration and rinsed with distilled water until no glutaraldehyde was detected in the filtrate. The determination of glutaraldehyde in the filtrate was performed as described by Boratynski and Zal (Boratyński and Zal 1990). The resulting MWCNT/Glu particles were then stored at room temperature until use.

MWCNT–OH was used as the starting material for glyoxyl activation because hydroxyl groups are required for the reaction with glycidol under alkaline conditions, leading to the formation of vicinal diols that can subsequently be converted into glyoxyl (aldehyde) groups. To generate glyoxyl groups, 1.0 gram of MWCNT-OH was mixed with 4 mL of NaOH/NaBH_4_ solution at 25 °C and then 1.0 mL of glycidol was slowly added at 5 °C. The mixture was incubated at 5 °C for 12 h in a temperature-controlled orbital shaker with agitation at 75 rpm. After that, the support was collected by vacuum filtration and rinsed with distilled water. Then, the support was treated with 10 mL of sodium metaperiodate solution (1 µmol) in water at 25 °C and the mixture was continuously stirred at 100 rpm using a magnetic stirrer for 2 h. The resulting support (MWCNT/Gly) was collected by vacuum filtration and rinsed with distilled water and stored at room temperature until use.

### Immobilization of RML

One gram of each support was suspended in 10 mL of immobilization buffer (50 mM sodium phosphate at pH 7.0 for MWCNT/Gen and MWCNT/Glu; 50 mM sodium carbonate at pH 10.0 for MWCNT/Gly) containing 1 mg protein/mL of RML. Each immobilization suspension was incubated at 25 °C for 2 h in a temperature-controlled orbital shaker with agitation at 100 rpm. Subsequently, the mixture was filtered under vacuum to collect the resulting immobilized RML preparations and washed with distilled water until no protein was detected in the filtrate at 280 nm. RML immobilized on MWCNT/Gen, MWCNT/Glu, and MWCNT/Gly were denoted as MWCNT/Gen@RML, MWCNT/Glu@RML, and MWCNT/Gly@RML (Fig. S1), respectively, and stored at 5 °C until use.

The immobilization yield and expressed activity were determined as described by Boudrant et al. (Boudrant et al. 2020).

$$\:Immobilization\:yield\:(\%=\frac{\left({P}_{i}-{P}_{s}\right)}{{P}_{i}}x100$$

where P_i_ and P_s_ are the initial added protein amount and protein amount in the supernatant, respectively.

BCA protein analysis kit was used to determine the initial added protein amount and protein amount in the supernatant.

$$\:Expressed\:activity\:\left(\%\right)=\frac{Activity\:of\:immobilized\:enzyme}{Total\:activity\:of\:enzyme\:offered\:for\:immobilization}$$

### Instrumental analysis

The successful immobilization of RML samples was verified through a series of physicochemical and morphological analyses. Fourier transform infrared spectroscopy (FTIR) was employed to identify functional groups and confirm chemical interactions, while scanning electron microscopy (SEM) was used for detailed visualization of surface topography. Energy-dispersive X-ray spectroscopy (EDS) coupled with SEM provided elemental mapping and compositional information.

### Activity assay

The catalytic activity of both free and immobilized RML was assessed using a *p*-nitrophenyl palmitate (*p*-NPP) based reaction system with a final volume of 1 mL (Gupta et al. 2002). Each assay contained 100 µL *p*-NPP (1.25 mM in isopropanol), 880 µL of Tris-HCl buffer (50 mM, pH 8.5) and 20 µL of enzyme solution (1 mg/mL) for the free enzyme, while 2.5 mg of immobilized biocatalyst was used for the immobilized preparations. Reactions were carried out for 10 min at their respective optimal temperatures, after which the release of *p*-nitrophenol (*p*-NP) was quantified by measuring the absorbance at 410 nm using an Agilent UV–Vis spectrophotometer. To terminate the reaction, 250 µL of sodium carbonate (0.1 M in dH_2_O) was added. One unit (U) of lipase activity is defined as the amount of enzyme that liberates 1 µmol of *p*-NP per minute under the assay conditions.

### Biochemical properties of free and immobilized RML variants

#### Optimum pH and temperature

The optimal pH values of the free RML and its immobilized forms were assessed by activity assays performed in distinct buffer systems. Measurements were conducted in 50 mM phosphate buffer (NaH_2_PO4–Na_2_HPO_4_, pH 6.0–7.0), and 50 mM Tris-HCl buffer (pH 7.5–9.0). Each reaction mixture (1 mL) contained 1.5 mM p-nitrophenyl palmitate (*p*-NPP) as the substrate. The mixtures were incubated at 45 °C with orbital shaking at 120 rpm for 10 min, followed by centrifugation at 10,000 rpm for 5 min. The supernatants were subsequently collected, and the absorbance was measured at 410 nm using a spectrophotometer. To determine the optimal temperature, assays were conducted at the previously identified optimal pH across a temperature range of 40–70 °C in 5 °C increments. The highest observed activity was defined as 100%, and all other values were expressed relative to this reference.

#### Kinetic parameters

Kinetic parameters, including *K*_*m*_, *k*_*cat*_, and catalytic efficiency (*k*_*cat*_/*K*_*m*_), were determined for both free RML and its immobilized preparations by monitoring the initial reaction rates at different *p*-NPP concentrations (0.125–1.5 mM). The reactions were performed at the optimal pH and temperature determined for each enzyme preparation. In all assays, equivalent amounts of enzyme were employed for both free RML and the immobilized preparations to ensure a reliable comparison of catalytic performance. The values were obtained by nonlinear regression analysis of data fitted to the Michaelis–Menten model. The catalytic efficiency ratios (CERs) were calculated by dividing the *k*_*cat*_/*K*_*m*_ value of each immobilized RML by that of the free RML. All assays were carried out under pH (7.5) and temperature (45–60 °C) conditions individually optimized for each enzyme form to ensure accurate kinetic evaluation.

#### Thermal stability

Thermal stability assays were conducted to evaluate the resistance of free RML and its immobilized derivatives under elevated temperature conditions, with the immobilized enzymes tested at their determined optimum temperatures. In each experiment, 5 U of free or immobilized RML were used. The enzyme preparations were incubated without substrate in 50 mM Tris-HCl buffer at their respective optimum temperatures for predetermined intervals (0, 1, 2, 4, 8, 16, and 24 h). At each time point, the residual catalytic activity was determined using standard assay procedures, with the initial activity set to 100%. The thermal inactivation profiles were subsequently analyzed to assess enzyme stability, and the deactivation rate constant (k_d_), stabilization factor (SF), and half-life (t₁/₂) were calculated from these data.

#### Thermogravimetric (TGA) analysis

TGA was performed to evaluate the thermal stability and organic content of free and immobilized RML samples using a Mettler Toledo TGA 3 + thermal analyzer, following methodologies similar to those previously reported (Özdemir et al. 2023, 2024; Tülek 2025). Measurements were conducted over a temperature range of 25 °C to 800 °C under a controlled atmosphere. All analyses were carried out using a high sensitivity microbalance with a resolution of 0.1 µg. Approximately 5 to 10 mg of each sample was placed in thermally and chemically compatible crucibles, and weight loss was continuously recorded as a function of temperature. The resulting thermograms were used to assess thermal stability, decomposition behavior, and relative organic content associated with enzyme immobilization on MWCNT-based supports.

### Enrichment of EPA and DHA from fish oil

Commercial fish oil (3 mL) was mixed with 4.5 mL n-hexane, 2.5 mL of 50 mM Tris-HCl buffer (pH 7.5), and free or immobilized lipase (2.5–7.5 U) to obtain a final reaction volume of 10 mL. The reaction mixture was vortexed for 3 min, then incubated at 50 °C with shaking at 250 rpm. Aliquots were withdrawn at predetermined intervals (12, 24, 48, and 60 h) and centrifuged at 9000 x g for 10 min at 4 °C. After centrifugation, 3 mL of the hexane phase containing the enriched ω-3 fatty acids (EPA and DHA) was carefully transferred into round-bottom flasks to avoid contamination by the oil layer. The solvent was removed using a rotary evaporator (Heidolph) operated at 35 °C and 180 rpm. The residue was then dissolved in 1 mL of methanol, vortexed for 3 min, filtered through a 0.45 μm syringe filter, and transferred to HPLC vials for analysis.

In experiments evaluating the effect of reaction time, 1 U of enzyme was used for each form, including free RML, MWCNT/Gen@RML, MWCNT/Glu@RML, and MWCNT/Gly@RML. Based on the optimal reaction time determined for each enzyme form, additional reactions were performed at enzyme loadings of 2.5, 5, and 7.5 U under the same experimental conditions described above, to assess the enrichment efficiency of EPA and DHA as a function of enzyme concentration. The optimal reaction time was determined from time-course hydrolysis experiments in which the accumulation of EPA and DHA was monitored at different reaction times, and the time point corresponding to the maximum EPA + DHA enrichment was selected as the optimal reaction time.

#### HPLC analysis

Quantification of EPA and DHA was performed using an HPLC system (Agilent 1260 Infinity Series) equipped with a diode array detector (DAD). Separation was achieved on an Eclipse XDB-C18 column (4.6 × 150 mm, 5 μm) maintained at 35 °C. The mobile phase consisted of solvent A (0.1% phosphoric acid in water, 30%) and solvent B (acetonitrile, 70%), delivered isocratically at a flow rate of 1.0 mL/min. The injection volume was 10 µL, and detection was carried out at 215 nm with a reference wavelength of 400 nm.

#### Reusability

Reusability experiments were conducted for immobilized lipase preparations using 1 U of enzyme under the same reaction conditions described above. Each reaction was carried out for 24 h at 50 °C and 250 rpm. Upon completion of the reaction, the mixtures were centrifuged at 9000 x g for 10 min at 4 °C. The recovered immobilized enzymes were sequentially rinsed with n-hexane to remove residual fish oil and other hydrophobic components, followed by distilled water. Subsequently, the immobilized enzymes were centrifuged again under the same conditions and directly reused in the next catalytic cycle. The supernatant obtained after centrifugation was subjected to HPLC analysis as described above. The immobilized enzymes were washed with distilled water, and then recentrifuged under the same conditions, and reused for subsequent cycles. This process was repeated for each reusability test to evaluate the operational stability and catalytic performance of the immobilized enzymes over multiple cycles.

### Statistical analysis

All experiments were carried out in triplicate, and the results are expressed as mean ± standard deviation (SD). Statistical analyses and graph generation were performed using GraphPad Prism 9 software. Differences were considered statistically significant at *p* < 0.05.

## Results and discussion

### Immobilization of RML

The immobilization pH plays a critical role in determining the reactivity of activating agents because it controls the protonation state of nucleophilic residues on the enzyme surface, particularly the ε-amino groups of lysine residues. Genipin reacts with primary amines through nucleophilic attack followed by heterocyclic crosslink formation and can proceed efficiently over a broader pH range, providing stable covalent linkages under relatively mild condition (Wahba 2024). For glutaraldehyde-activated supports, covalent attachment occurs mainly through Schiff base formation between aldehyde groups and amino groups of the enzyme. At low pH values, amino groups remain protonated and less nucleophilic, limiting covalent bonding. Near-neutral pH conditions enhance partial deprotonation of lysine residues, increasing their reactivity and facilitating enzyme–support coupling (Betancor et al. 2006; Vescovi et al. 2016; Okura et al. 2020; Miguez et al. 2023). In contrast, glyoxyl-activated supports typically require strongly alkaline conditions (around pH 10) to promote extensive deprotonation of lysine residues and enable multipoint covalent attachment, which contributes to enhanced enzyme stabilization (Mateo et al. 2005). The immobilization of RML on MWCNT/Gen, MWCNT/Glu, and MWCNT/Gly was studied at different pH values, and the corresponding immobilization yields and expressed activities are presented in Table S1. Across all supports and tested conditions, high immobilization yields (73–86%) were obtained indicating that all activation strategies were effective in providing functional groups that covalently immobilize RML molecules. The immobilization yield of RML on MWCNT/Glu reached a maximum of 81.1% at pH 7.0, likely due to optimal reactivity of aldehyde groups on the support with amino residues of RML. Similarly, MWCNT/Gen exhibited a consistently high immobilization yield (~ 78–84%) across the studied pH range (5.0–10.0), demonstrating the stable crosslinking capability of genipin. MWCNT/Gly also achieved high binding efficiency (80–86%), suggesting that the aldehyde groups generated through oxidation of vicinal diols effectively interacted with the enzyme’s amino groups, particularly under alkaline conditions that favour Schiff base formation.

The immobilization pH also significantly influenced the expressed activity of the immobilized RML derivatives, likely due to its effect on enzyme orientation and the extent of enzyme–support interactions during covalent attachment. The higher activity of MWCNT/Gen@RML observed at acidic conditions suggests that milder immobilization environments help preserve the native conformation of the enzyme and maintain better accessibility of the active site. The activity decreased from 59.5% at pH 5.0 to 25.5% at pH 10.0 for MWCNT/Gen@RML, indicating partial conformational restriction or active site distortion at alkaline pH. Meanwhile, MWCNT/Glu@RML achieved the highest expressed activity (42.9%) at pH 7.0, suggesting that MWCNT/Glu favor efficient enzyme-support coupling under near-neutral conditions, but may lead to over-crosslinking or increased structural rigidity under more acidic or basic conditions (Özdemir et al. 2023). MWCNT/Gly@RML showed its highest expressed activity (38.4%) at pH 10.0, compared with 27.2% and 25.9% at pH 7.0 and 5.0, respectively. This trend suggests that immobilization at alkaline pH enhances the activation of surface aldehyde groups of MWCNT/Gly and promotes multipoint covalent binding, which may stabilize the enzyme structure without significantly altering its active site geometry (Jun et al. 2019; Almeida et al. 2021). Overall, the results reveal that the activation chemistry of the support and the immobilization pH strongly influence both enzyme loading and catalytic performance. MWCNT/Gly, particularly at alkaline pH, provided a favourable microenvironment for RML attachment and retained the highest catalytic efficiency. In contrast, glutaraldehyde and genipin activations, while achieving comparable immobilization yields, resulted in higher expressed enzyme activities, possibly due to much favourable enzyme orientation (Pinheiro et al. 2023; Akbulut et al. 2025).

**Fig. 1:**
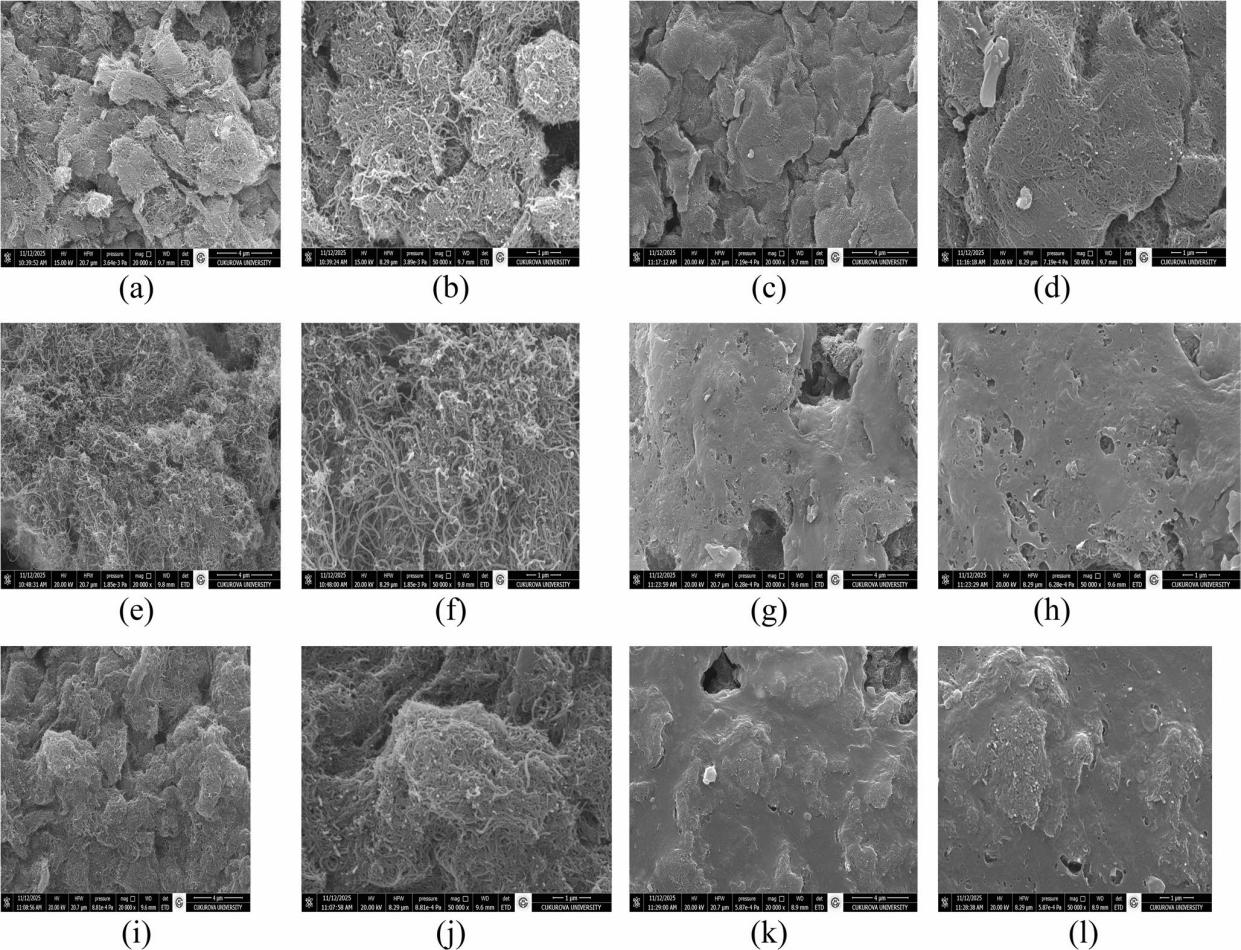
SEM micrographs illustrating the surface morphology of MWCNT based materials before and after immobilization of RML. (**a**, **b**) MWCNT/Gen; (**c**, **d**) MWCNT/Gen@RML; (**e**, **f**) MWCNT/Glu; (**g**, **h**) MWCNT/Glu@RML; (**i**, **j**) MWCNT/Gly; (**k**, **l**) MWCNT/Gly@RML. The images reveal the morphological differences between the functionalized MWCNT materials and the corresponding enzyme immobilized biocatalysts. Magnifications: 20,000× (**a**, **c**, **e**, **g**, **i**, **k**) and 50,000× (**b**, **d**, **f**, **h**, **j**, **l**)

The morphological alterations observed in RML immobilized on differently modified MWCNTs are presented in the SEM images of Fig. 1. Following immobilization, the MWCNT surfaces exhibited noticeable morphological changes, including the appearance of coating-like structures and partial surface coverage, which are consistent with enzyme attachment on the nanotube network (Jun et al. 2019; Özdemir et al. 2024; Tülek et al. 2025). Complementary elemental mapping obtained by SEM–EDS analysis is shown in Fig. S2, further confirming the successful attachment of RML to the modified supports. Prior to immobilization, the spectra of all MWCNT materials were dominated by C and O signals, with minor N signals attributable to surface modifications (Fig. S2a–c). In contrast, spectra recorded after immobilization showed the appearance of S in all samples (Fig. S2d–f). The presence of an S signal exclusively in the post-immobilization spectra represents a characteristic elemental marker originating from sulfur-containing amino acids in the protein, thereby confirming the deposition of the enzyme on the support. Additionally, the increase in N content after immobilization is indicative of the proteinaceous nature of the attached biomolecules (Tülek et al. 2023; Grmasha et al. 2024). Taken together, these elemental changes provide clear analytical evidence that RML was successfully immobilized on all MWCNT-based supports.

The FTIR spectra indicated the successful immobilization of RML on all three modified MWCNT supports, a conclusion further supported by comparison with the spectrum of the free enzyme. For MWCNT/Gen@RML (Fig. S3a), the emergence of characteristic protein-related bands, including the broad N–H stretching region around 3300 cm^− 1^ (Moreira et al. 2020), and the appearance of amide I and amide III bands at approximately 1608 and 1265 cm^− 1^, respectively, suggested the presence of the enzyme on the carrier surface, while the attenuation and deformation of the genipin carbonyl band at 1722 cm^− 1^ implied its involvement in interactions between RML and the support (Abdul Manan et al. 2018; Zhang et al. 2020; Moreira et al. 2020). A similar pattern was observed for MWCNT/Glu@RML (Fig. S3b): the appearance of amide bands, broadening of the N–H region, and reduction of the aldehyde C = O band at approximately 1722 cm^− 1^ were consistent with covalent attachment mediated by glutaraldehyde (Jun et al. 2019; Özdemir et al. 2024). In the case of MWCNT/Gly@RML (Fig. S3c), weaker attenuation of the carbonyl band and less pronounced changes in the amide regions suggested a mixed immobilization mechanism involving partial covalent bonding together with physical adsorption. The FTIR spectrum of free RML (Fig. S3d) exhibited typical protein features, including a broad N–H stretching band at approximately 3318 cm^− 1^ and prominent amide I and amide III bands at 1638 and 1275 cm^− 1^, respectively (Özdemir et al. 2024). These features corresponded well with the bands observed in the immobilized samples and supported the interpretation that the spectral changes originated from enzyme attachment (Özdemir et al. 2024).

### Biochemical characterization

**Fig. 2:**
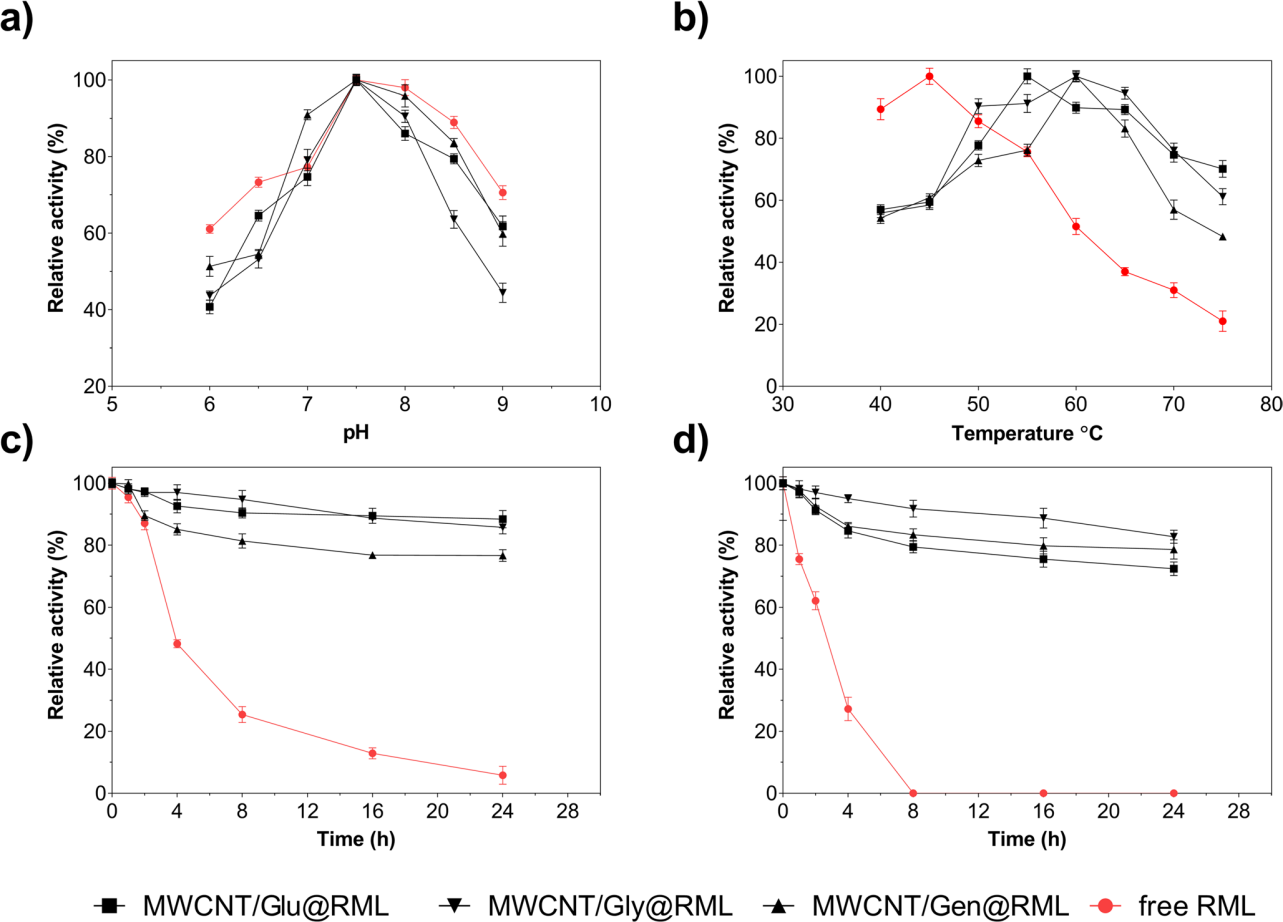
Effect of pH (**a**), temperature (**b**), and thermal stability at 55 °C (**c**) and 60 °C (**d**) on free and immobilized RML variants. The effect of pH was conducted in 50 mM phosphate buffer (pH 6.0–7.0), and 50 mM Tris-HCl buffer (pH 7.5–9.0) at 45 °C. Effect of temperature was studied in the temperature range of 40–70 °C at pH 7.5. Thermal stability experiments were investigated at 55 °C and 60 °C for 24 h

All RML preparations exhibited a bell-shaped pH–activity profile, with the highest catalytic performance consistently observed at pH 7.5 (Fig. 2a). Both the free enzyme and the MWCNT/Gen@RML biocatalyst maintained 85–90% of their maximal activity between pH 7.0 and 8.5, indicating that the immobilization of RML via genipin provides a more adaptable microenvironment that stabilizes the catalytic residues (Esparza-Flores et al. 2023; Tülek 2025). In contrast, MWCNT/Glu@RML and MWCNT/Gly@RML displayed narrower operational pH windows and experienced substantial activity losses above pH 8.0, suggesting that the stronger covalent interactions introduced by aldehyde groups impose greater restrictions on conformational flexibility (Cowan and Fernandez-Lafuente 2011; Alagöz et al. 2021). Previous reports align with these observations. Moreira et al. (2020) demonstrated that RML immobilized on APTES-coated magnetic nanoparticles (Fe₃O₄@APTES-RML) exhibited an optimal pH of 7.0, whereas Ghide et al. (2022) showed that magnetic MWCNT-supported RML reached maximal activity at pH 7.0 and exhibited enhanced pH stability after immobilization. Similarly, Alagöz et al. (2021) reported that RML immobilized on Si–COOH, Si–Glu, and MWCNT–COOH supports achieved maximum activity at pH 7.5, with immobilized forms displaying markedly improved stability across the evaluated pH range relative to the free enzyme.

A comparison of the temperature–activity profiles revealed clear distinctions among the RML preparations (Fig. 2b). The free enzyme exhibited maximum activity at 45 °C, followed by a pronounced decline at higher temperatures, retaining only about 30% activity at 70 °C. In contrast, MWCNT/Glu@RML and MWCNT/Gly@RML exhibited optimal activity at 55 °C and retained 74% and 76% of that activity at 70 °C, respectively, demonstrating substantially improved thermal stability. MWCNT/Gen@RML displayed the highest optimum temperature at 60 °C and retained 57% activity at 70 °C, thereby identifying it as the most thermally robust variant among the immobilized forms. These results indicate that immobilization effectively enhances RML’s resistance to temperature-induced denaturation. Consistent with these findings, Alagöz et al. (2021) examined RML immobilized on Si–COOH, Si–Glu, and MWCNT–COOH supports, and reported that free RML, RML@Si–COOH, RML@MWCNT–COOH, and RML@SCNT–Glu all reached their maximum activity at 40 °C, whereas RML@Si–Glu exhibited a shifted optimum at 50 °C. A similar trend was observed for immobilized *Candida rugosa* lipase on cobalt-doped magnetic MWCNTs functionalized with aminated polydopamine. In the study, the free enzyme exhibited an optimum temperature of 40 °C, while the immobilized form reached its maximum activity at 45 °C and retained nearly 70% activity at 70 °C, compared with less than 50% for the free enzyme, indicating a substantial enhancement in thermal stability (Asmat et al. 2019). Sulym et al. (2021) reported complementary findings for *Candida antarctica* lipase B immobilized on pristine and PDMS-modified MWCNTs. All preparations showed an optimum temperature of 30 °C; however, the free enzyme rapidly lost activity outside this narrow range, whereas the immobilized systems preserved over 60% relative activity across the wider interval of 20–70 °C, with the MWCNTs/PDMS-100 support offering the best performance. Taken together, these studies underscore that immobilization on nanostructured carbon-based supports consistently improves the thermal behavior of lipases, a trend consistent with the enhanced temperature tolerance observed in immobilized RML variants.

At both 55 °C and 60 °C, the thermal stability profiles revealed that immobilization markedly protected RML against heat-induced deactivation, though the extent varied depending on the spacer arm used (Fig. 2c and d). At 55 °C, the immobilized derivatives maintained nearly constant activity throughout the 24-h incubation, while free RML lost more than 90% of its initial activity within the same period. Among the immobilized forms, MWCNT/Glu@RML and MWCNT/Gly@RML displayed the most stable behaviour, preserving approximately 85–90% of their residual activity after 24 h; MWCNT/Gen@RML followed, retaining around 75–80%. When the incubation temperature was increased to 60 °C, activity decay became more pronounced, but the immobilized RMLs still showed remarkable resistance to thermal inactivation compared with free RML, which was almost completely inactivated within 8 h. MWCNT/Gly@RML exhibited the highest retention, maintaining over 80% activity after 24 h, whereas MWCNT/Gen@RML and MWCNT/Glu@RML remained at about 70–75%. Fan et al. (2017) immobilized *Burkholderia cepacia* lipase (BCL) on MWCNTs that were rendered magnetic by iron oxide loading and subsequently functionalized with PAMAM dendrimers, via a covalent attachment strategy. Their results showed that the BCL-mMWCNTs-G3 system exhibited markedly enhanced thermal stability compared with the free enzyme. After 1 h of incubation at 65 °C, free BCL retained only a small fraction of its activity, whereas the immobilized form preserved more than 86% activity, highlighting the substantial improvement in high-temperature stability achieved through immobilization. Moguei et al. (2022) immobilized *Thermomyces lanuginosus* lipase onto epoxy-functionalized MWCNT-OH and assessed its thermal behavior after 2 h of incubation. The immobilized enzyme retained full activity at 55 °C, whereas the free enzyme retained approximately 91% activity. At 75 °C, the free lipase showed 25.6% residual activity, whereas the immobilized preparation maintained 53.2% residual activity, demonstrating its superior thermal tolerance. Asmat et al. (2019) immobilized *C. rugosa* lipase onto Co-doped magnetic MWCNTs functionalized with aminated polydopamine and demonstrated markedly enhanced thermal stability compared to the free enzyme. After incubation at 60 °C for 1 h, the residual activity of free CRL dropped to below 20%, whereas the immobilized preparation retained approximately 55–60% of its initial activity. Moreover, the free enzyme exhibited maximal activity at 40 °C, while the immobilized CRL displayed optimal activity at 45 °C, confirming the improved thermal resistance conferred by the nano-support. In line with existing literature, the pronounced thermal resilience observed for MWCNT/Glu@RML, MWCNT/Gly@RML, and MWCNT/Gen@RML underscores the stabilizing capacity of MWCNT-based supports, indicating that each spacer arm provides a distinct yet consistently beneficial contribution to enzyme performance under high-temperature conditions.

Thermal stability analysis indicated that immobilization of RML on differently modified MWCNTs was associated with improved resistance to thermal inactivation at both 55 °C and 60 °C (Table S2). Free RML exhibited relatively high k_d_ values of 0.12 h⁻^1^ at 55 °C and 0.32 h⁻^1^ at 60 °C, corresponding to t_1/2_ values of 5.7 and 2.1 h, respectively, suggesting limited intrinsic thermal durability under the tested conditions. In contrast, all immobilized systems showed notably reduced k_d_ values and prolonged t_1/2_, reflecting a general tendency toward decreased thermal deactivation rates upon immobilization. Among the immobilized preparations, MWCNT/Glu@RML displayed a lower k_d_ value (0.0048 h⁻¹) and a longer t_1/2_ (144.6 h) at 55 °C, whereas MWCNT/Gly@RML exhibited comparatively lower k_d_ (0.0073 h⁻¹) and longer t_1/2_ (94.8 h) at 60 °C. MWCNT/Gen@RML also demonstrated enhanced stability relative to free RML, although its performance appeared less pronounced when compared with the glyoxyl and glutaraldehyde coupling agents, particularly at 55 °C. The stabilization factor values were consistent with these observations, with immobilized forms showing SF values ranging from 11.4 to 25.3 at 55 °C and from 25.5 to 44.4 at 60 °C. Notably, the SF value of MWCNT/Gly@RML at 60 °C was markedly higher than those of the other coupling agents, indicating that this preparation may retain activity more effectively under elevated temperature conditions. Overall, these kinetic parameters suggest that immobilization exerts a substantial influence on the thermal inactivation behavior of RML, and that the magnitude of this effect varies depending on the nature of the support used.

The TGA profiles of MWCNT supports before and after RML immobilization are presented in Fig. S4. In all immobilized samples, higher total mass loss values were observed compared with the corresponding bare supports, indicating the introduction of additional organic content associated with enzyme attachment. The weight loss observed below approximately 150 °C was small for both the bare and immobilized samples and was attributed mainly to the removal of physically adsorbed moisture and surface-bound volatile components. Between approximately 200 °C and 600 °C, the immobilized samples exhibited markedly greater mass loss than the corresponding supports, consistent with the thermal decomposition of proteinaceous material. In particular, MWCNT/Gen@RML and MWCNT/Gly@RML exhibited more pronounced weight loss in this region, suggesting higher enzyme loading on these supports (Yuan et al. 2023; Bilal et al. 2024). The thermogram of free RML revealed rapid and extensive mass loss beginning near 150 °C and reaching near-complete decomposition by 400 °C, confirming the purely organic nature of the enzyme and its limited thermal stability. In contrast, all immobilized enzyme preparations retained a substantial residual mass beyond 700 °C, reflecting the high thermal resistance of the MWCNT matrix (Dutra Rosolen et al. 2017; Massoumi et al. 2019). The shift in degradation behavior and the increased mass loss in immobilized samples, relative to bare supports, clearly verified successful enzyme incorporation and an apparent enhancement of thermal robustness.

According to Table S3, the kinetic parameters of free and immobilized RML variants demonstrate that immobilization markedly affected the catalytic characteristics of RML. The free RML exhibited a *k*_*cat*_ value of 43.4 min^− 1^. In contrast, the MWCNT/Glu@RML variant displayed the highest *k*_*cat*_ of 62.7 min^− 1^, suggesting that under suitable conditions, immobilization can further enhance enzymatic activity. A similar trend was observed for the calculated *V*_*max*_ values, where MWCNT/Glu@RML exhibited the highest maximum reaction rate (1.44 U/mg prot), followed by free RML (0.82 U/mg prot), MWCNT/Gly@RML (0.76 U/mg prot), and MWCNT/Gen@RML (0.35 U/mg prot). These results suggest that the glutaraldehyde-linked immobilization strategy contributes to improved catalytic turnover under the tested conditions. The *K*_*m*_ values of free and immobilized RML derivatives ranged from 2.94 to 3.42 mM, indicating that substrate-binding affinity was not significantly influenced by immobilization. In terms of catalytic efficiency (*k*_*cat*_/*K*_*m*_), MWCNT/Glu@RML exhibited the highest value, 19.2 mM^− 1^ min^− 1^, confirming the beneficial effect of glutaraldehyde-mediated immobilization on catalytic performance. MWCNT/Gen@RML and MWCNT/Gly@RML variants showed lower catalytic efficiencies than those of the free enzyme. These findings highlight that the applied immobilization strategy plays a pivotal role in improving enzyme stability and catalytic potential. The observed differences among the immobilized systems can be attributed to variations in enzyme–support interactions, which affect enzyme rigidity, orientation, and substrate accessibility. In particular, glyoxyl-based immobilization promotes multipoint attachment, leading to increased rigidity, whereas genipin and glutaraldehyde provide a more balanced structural environment that preserves conformational flexibility. This balance facilitates substrate diffusion and active site accessibility, thereby contributing to the observed differences in catalytic performance and selectivity. These effects are consistent with the proposed mechanistic scheme (Fig. S1), which illustrates how different coupling chemistries generate distinct structural environments that govern enzyme dynamics and catalytic behaviour. CER values for MWCNT/Gen@RML, MWCNT/Glu@RML, and MWCNT/Gly@RML were approximately 0.7, 1.4, and 0.8, respectively. These results emphasize that the chosen coupling agent is a crucial determinant of the enzyme’s overall performance. Asmat et al. reported a clear improvement in the kinetic parameters of CRL after immobilization onto PDA@Co-MWCNTs. The immobilized enzyme exhibited a markedly lower *K*_*m*_ (11.2 mM) than the free CRL (19.5 mM), indicating enhanced substrate affinity. Although *V*_*max*_ decreased slightly upon immobilization, the catalytic efficiency (*V*_*max*_/*K*_*m*_) increased substantially, from 0.53 to 0.91 min⁻¹ (Asmat et al. 2019). In another study, Alagöz et al. immobilized *R. miehei* lipase onto Si-COOH, Si-Glu, MCNT-COOH, and SCNT-Glu supports and determined the apparent kinetic parameters toward p-NPA, reporting *K*_*m*_ values of 5.5, 5.4, 4.9, 3.7, and 5.3 mM and *V*_*max*_ values of 1.3, 0.9, 1.1, 0.6, and 0.5 U/mg, respectively; only RML@MCNT-COOH showed a lower *K*_*m*_, indicative of higher substrate affinity (Alagöz et al. 2021). The enhanced *k*_*cat*_ and catalytic efficiency of MWCNT/Glu@RML confirm that an optimally engineered CNT microenvironment can outperform previously reported CNT-lipase systems, underscoring the considerable potential of our design for high-performance biocatalysis.

### HPLC analysis for enrichment of fish oil

The HPLC analysis achieved baseline separation and accurate quantification of EPA and DHA under the described chromatographic conditions. As shown in Fig. S5, distinct and well-resolved peaks were obtained at retention times of 11.25 min (EPA) and 15.53 min (DHA). The calibration curves exhibited excellent linearity within the tested concentration range (0–10 µg/mL), with correlation coefficients (R²) of 0.99999 for EPA and 1.00000 for DHA, confirming the high precision and reliability of the quantification method. The slope values (99.36 for EPA and 85.94 for DHA) indicate a strong detector response and sensitivity at 215 nm. These results demonstrate that the developed HPLC method provides robust, reproducible, and sensitive quantification of EPA and DHA in enzymatically enriched samples. Representative HPLC chromatograms of the post-catalytic reaction mixtures are provided in Fig. S6 to illustrate the typical EPA and DHA peaks detected in the enzymatically hydrolyzed samples.

**Fig. 3:**
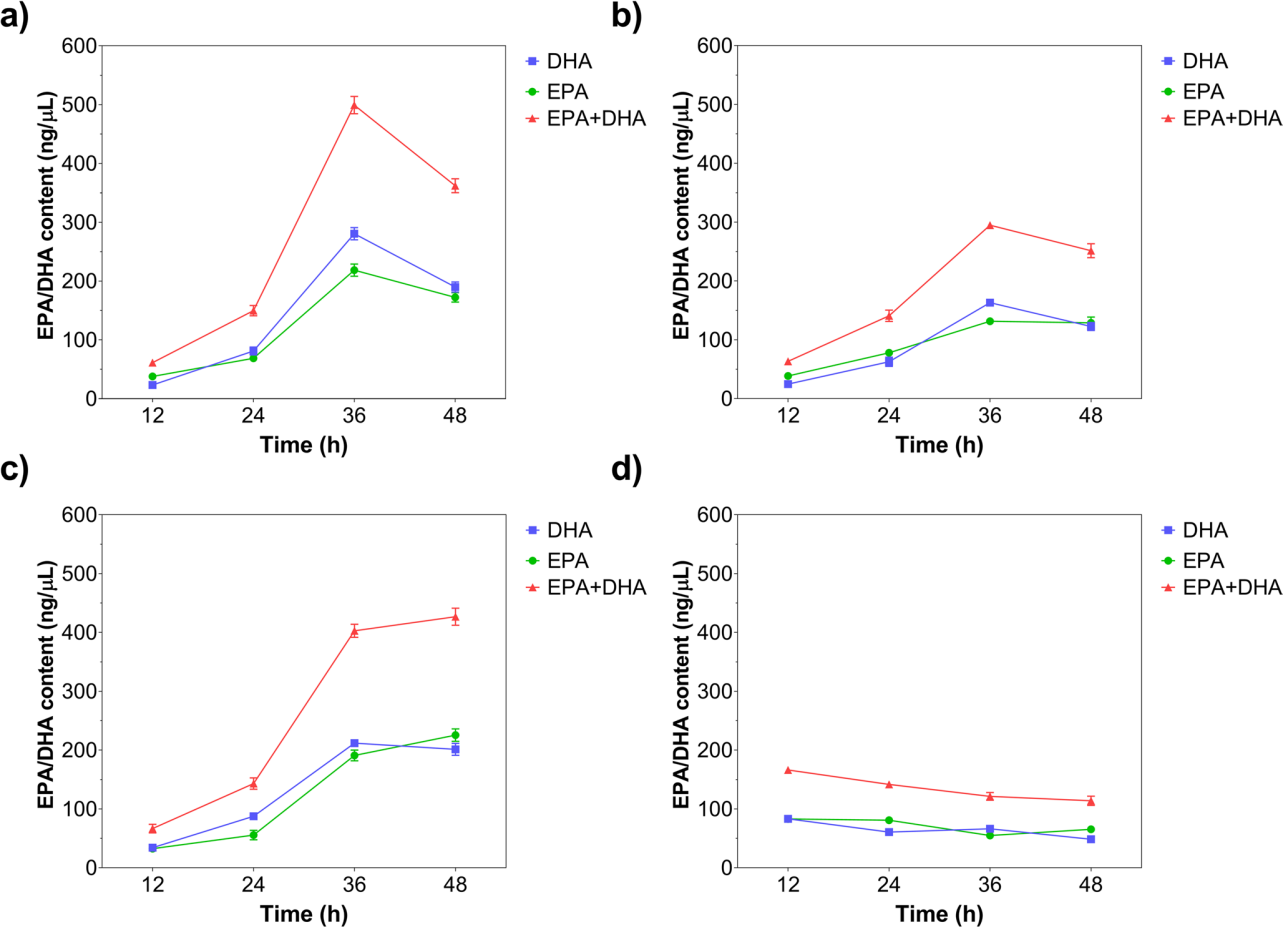
Time dependent formation of EPA and DHA during enzymatic hydrolysis of fish oil catalyzed by immobilized and free RML. (**a**) MWCNT/Gen@RML, (**b**) MWCNT/Glu@RML, (**c**) MWCNT/Gly@RML, and (**d**) free RML. The concentrations of DHA, EPA, and their combined amount (EPA + DHA) were quantified at different reaction times (12–48 h) using HPLC analysis. Error bars indicate SD

Time-course HPLC quantification of EPA + DHA is presented in Fig. 3. The EPA + DHA concentration increased from 61.25 ng/µL to 499.26 ng/µL when the hydrolysis time was increased from 12 h to 36 h for MWCNT/Gen@RML. A further increase in hydrolysis time to 48 h led to a decrease in the EPA + DHA amount to 362.12 ng/µL (Fig. 3a). Similarly, MWCNT/Glu@RML accumulated greater amounts of EPA + DHA when the hydrolysis time was increased from 12 h to 36 h. However, further increases in hydrolysis time did not significantly change the EPA + DHA content. After 48 h, the amount of EPA + DHA was 426.88 ng/µL (Fig. 3b). For MWCNT/Gly@RML, the highest EPA + DHA concentration was 294.83 ng/µL after 36 h of hydrolysis. Increasing the reaction time to 48 h led to a decrease in EPA + DHA concentration to 251.57 ng/µL (Fig. 3c). In contrast, the highest EPA + DHA concentration was obtained at 166.23 ng/µL for the free RML after a 12-h reaction. Increasing reaction time led to a decrease in EPA + DHA concentration and the concentration was 113.98 ng/µL after 48 h, indicating that extended incubation reduces product yield, likely due to enzyme deactivation or product degradation (Fig. 3d). In addition, the observed decrease in EPA + DHA content at prolonged reaction times may also be associated with secondary hydrolysis of glyceride intermediates. As the reaction proceeds, lipase can further hydrolyze diacylglycerols and monoacylglycerols formed during the initial stages of hydrolysis, which may lead to partial degradation or redistribution of polyunsaturated fatty acids (Yu et al. 2024). Moreover, prolonged exposure of highly unsaturated fatty acids such as EPA and DHA to reaction conditions may increase their susceptibility to oxidation or non-selective hydrolysis, thereby reducing the apparent enrichment level over time (Kotsoni et al. 2024).

**Fig. 4:**
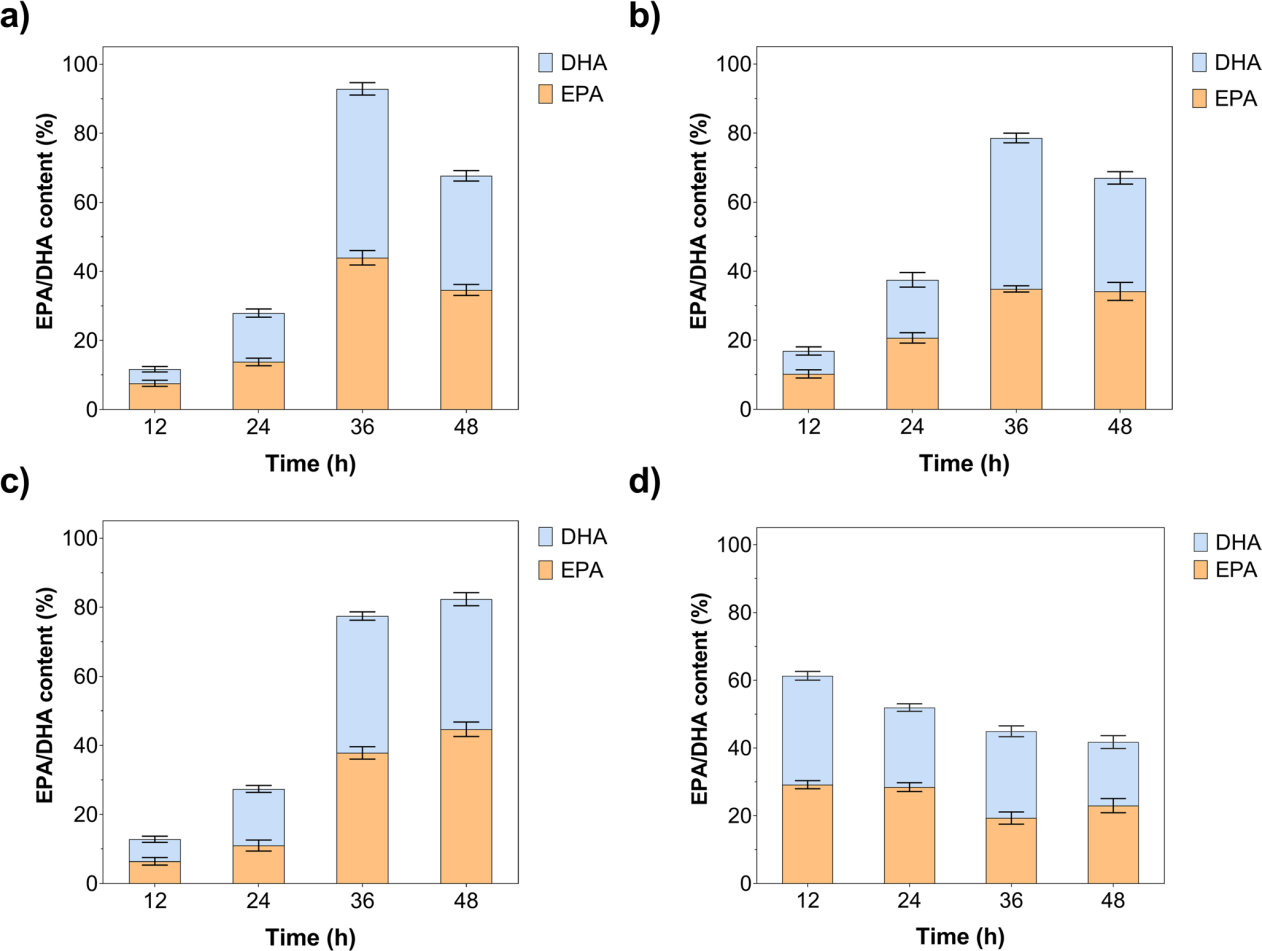
Time dependent changes in EPA and DHA content (%) during enzymatic hydrolysis of fish oil catalyzed by immobilized and free RML. (**a**) MWCNT/Gen@RML, (**b**) MWCNT/Glu@RML, (**c**) MWCNT/Gly@RML, and (**d**) free RML. Values are shown for different reaction times (12–48 h). Error bars indicate SD

Figure 4 demonstrates the time-dependent formation of EPA + DHA (%) during the hydrolysis of fish oil catalyzed by free and immobilized RML derivatives. Lipase catalyzes the hydrolysis of triacylglycerols through a serine-based catalytic triad, generating a transient acyl enzyme intermediate during the reaction. Due to its positional selectivity, the enzyme preferentially hydrolyzes fatty acids located at the sn-1 and sn-3 positions of the glycerol backbone, whereas fatty acids at the sn-2 position are hydrolyzed more slowly. Since long-chain polyunsaturated fatty acids such as EPA and DHA are frequently retained in this position, the remaining glyceride fraction becomes progressively enriched with these fatty acids during the course of hydrolysis (Akanbi et al. 2013; Yan et al. 2025). For MWCNT/Gen@RML, EPA + DHA yields were 7.61% and 4.07%, respectively, after 12 h of reaction (Fig. 4a). The highest EPA and DHA yields reached 43.96% and 48.89%, respectively, at 36 h for the MWCNT/Gen@RML system. According to the results, the EPA/DHA ratio was calculated to be 0.78. For MWCNT/Glu@RML, EPA yield increased from 6.45% at 12 h to 44.69% at 36 h. The corresponding increase in DHA ranged from 6.65 to 39.61% (Fig. 4b). For MWCNT/Gly@RML, the EPA + DHA (%) peaked at 36 h of reaction (EPA: 34.88%; DHA: 43.70%) and decreased slightly by 48 h (EPA: 34.16%; DHA: 32.84%) (Fig. 4c). In contrast, free RML exhibited a linear decrease in EPA + DHA content (%) (EPA 29.19% → 23.01%; DHA 32.13% → 18.75%) (Fig. 4d). When EPA + DHA totals are considered, MWCNT/Gen@RML achieved the highest EPA + DHA yield at 36 h (92.85%); MWCNT/Glu@RML reached a yield of 82.33% at 48 h; and MWCNT/Gly@RML peaked at 78.58% at 36 h, whereas EPA + DHA yield for free RML decreased from 61.32% at 12 h to 41.76% at 48 h. Overall, the immobilized RML derivatives—particularly MWCNT/Gen@RML—significantly enhanced EPA + DHA formation compared with free RML, demonstrating superior stability and catalytic efficiency during prolonged reaction times. The improved performance of immobilized derivatives may also arise from enhanced interfacial activation and the promotion or stabilization of the open-lid conformation of lipase on the hydrophobic MWCNT surface, which facilitates substrate access to the active site and improves catalytic turnover (Guimarães et al. 2023). Comparison at equivalent reaction times further emphasized the catalytic advantage conferred by immobilization. At 36 h, the total EPA + DHA yields of MWCNT/Gen@RML, MWCNT/Glu@RML, and MWCNT/Gly@RML were approximately 4.1, 3.3, and 2.4 folds higher than that of free RML. Mohammadi et al. (2015) showed that the formation rate of PUFA (EPA + DHA) content increased in the initial stage of the reaction, and then slightly decreased after 10 h of reaction for both free and immobilized RML derivatives.

**Fig. 5:**
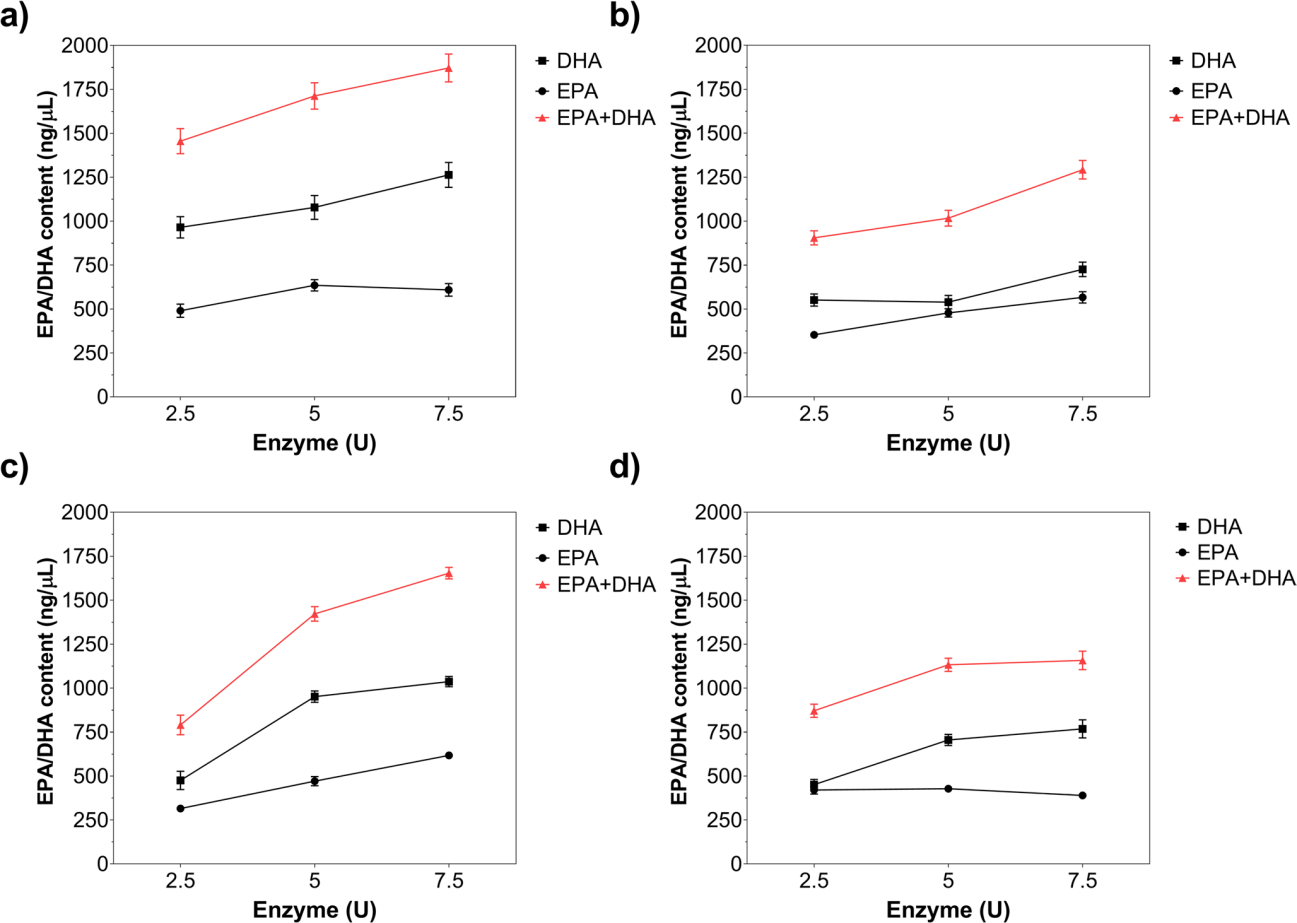
Effect of enzyme loading on EPA and DHA enrichment during enzymatic hydrolysis of fish oil. (**a**) MWCNT/Gen@RML, (**b**) MWCNT/Glu@RML, (**c**) MWCNT/Gly@RML, and (**d**) free RML. DHA, EPA, and total EPA + DHA concentrations (ng/µL) were determined at different enzyme activities (2.5–7.5 U) under the optimal reaction time established for each enzyme form at 50 °C. Error bars indicate SD

The effect of enzyme loading (2.5–7.5 U) on total EPA + DHA concentrations (ng µL⁻¹) is demonstrated in Fig. 5. EPA + DHA concentration increased from 1455.2 to 1871.9, primarily attributable to DHA accumulation (from 964.8 to 1263.2), while maintaining DHA-biased selectivity (EPA/DHA ≈ 0.48–0.59) for MWCNT/Gen@RML. MWCNT/Glu@RML exhibited a pronounced loading response, with the total concentration increasing from 790.6 to 1653.9 ng/µL, accompanied by substantial increases in both EPA and DHA. In contrast, MWCNT/Gly@RML exhibited a moderate increase in concentration, rising from 905.2 to 1292.5 ng/µL, accompanied by steady EPA accumulation and only minor fluctuations in DHA levels. Accordingly, the combined EPA + DHA concentration increased modestly from 871.3 to 1157.7 ng/µL, reflecting limited EPA formation and a gradual accumulation of DHA by the free RML. At 7.5 U, MWCNT/Gen@RML exhibited the highest total product yield (1871.9), followed by MWCNT/Glu@RML (1653.9), MWCNT/Gly@RML (1292.5), and free RML (1157.7), corresponding to approximately 1.62-, 1.43-, and 1.12-fold enhancements relative to the free enzyme. Overall, immobilization markedly enhances product formation and favors DHA selectivity, with 7.5 U identified as the optimal loading for maximum productivity.

**Fig. 6:**
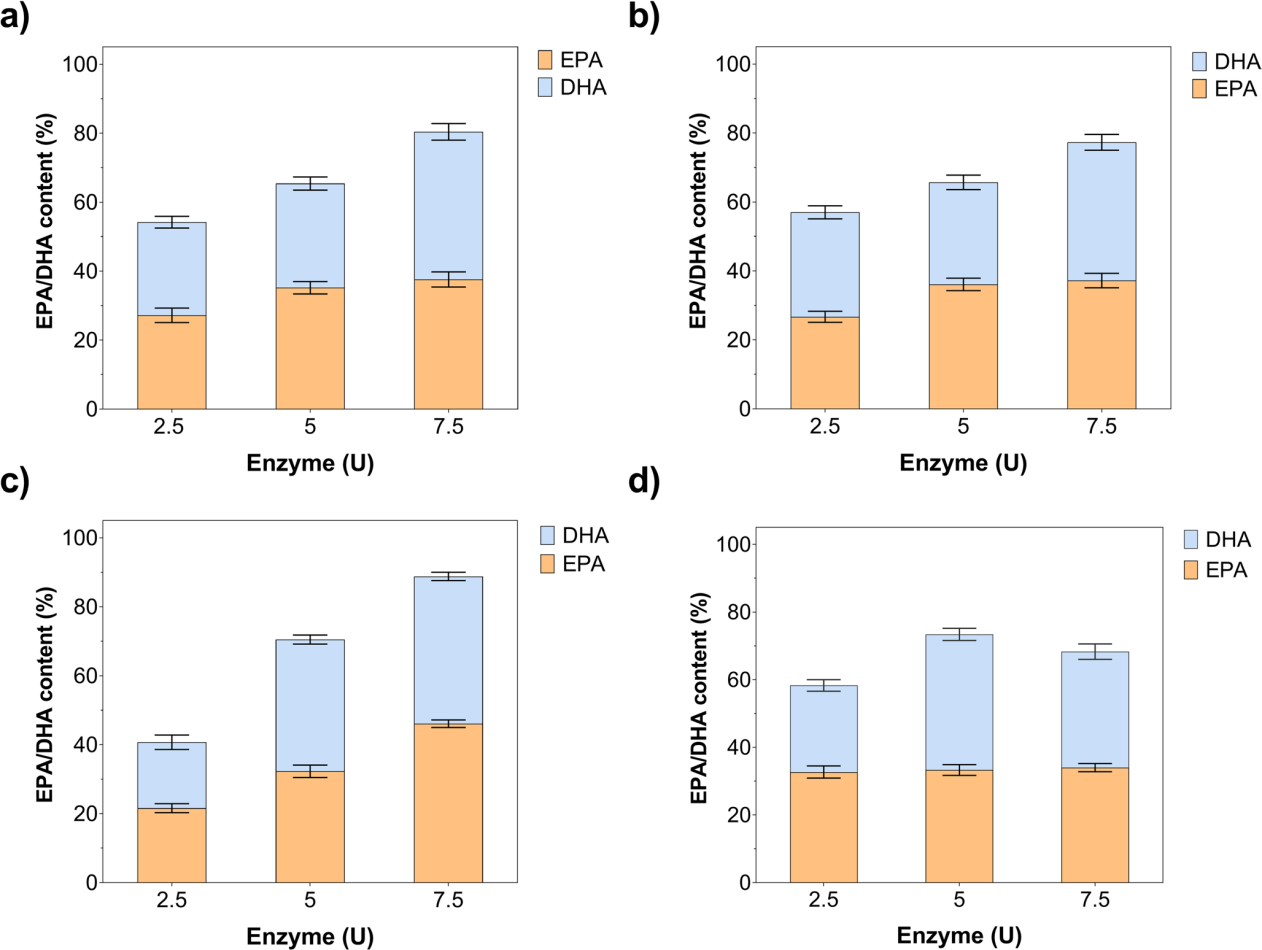
Effect of enzyme loading on EPA and DHA content (%) during enzymatic hydrolysis of fish oil. (**a**) MWCNT/Gen@RML, (**b**) MWCNT/Glu@RML, (**c**) MWCNT/Gly@RML, and (**d**) free RML. The relative percentages of EPA and DHA were determined at different enzyme activities (2.5–7.5 U) under the optimal reaction time established for each enzyme form at 50 °C. Error bars indicate SD

Across enzyme loadings of 2.5–7.5 U, all immobilized RML preparations exhibited a consistent increase in total EPA + DHA yield, whereas free RML plateaued at 5 U and declined thereafter (Fig. 6). At 7.5 U, the total yield followed the order MWCNT/Glu@RML (88.8%) > MWCNT/Gen@RML (80.4%) > MWCNT/Gly@RML (77.3%) > free RML (68.3%). MWCNT/Glu@RML displayed the strongest response to enzyme loading and shifted toward EPA selectivity (EPA/DHA = 1.08), while MWCNT/Gen@RML and MWCNT/Gly@RML favored DHA formation (EPA/DHA = 0.88 and 0.93, respectively). The decline in free RML activity at higher loadings suggests mass-transfer limitations or product inhibition. In addition, increasing enzyme loading may reduce the apparent selectivity of the hydrolysis reaction. At higher enzyme concentrations, a larger number of active sites become available simultaneously, which can promote more extensive and less selective hydrolysis of triacylglycerols. Under such conditions, the positional selectivity of lipase toward the sn-1 and sn-3 positions may become less pronounced, allowing further hydrolysis of intermediates and partial degradation of previously enriched polyunsaturated fatty acids. As a result, the relative EPA/DHA distribution may change at higher enzyme loadings (Yousefi et al. 2020; Yu et al. 2025). Overall, glutaraldehyde-mediated immobilization most effectively enhances the synthesis of EPA and DHA, while modulating selectivity toward EPA.

Table 1 shows the performances of different immobilized lipase preparations for the enrichment of EPA and DHA. Immobilized RML, *B. cepacian* and *B. subtilis* lipases generally favored EPA enrichment, achieving increases of 33.2% and 18.7%, respectively, while showing minimal effects on DHA enrichment (Ahrari et al. 2022; Liu et al. 2024; Verma et al. 2019). In contrast, co-immobilized *T. lanuginosus* and *C. antarctica* lipases on chitosan carriers provided balanced enrichment, raising total EPA + DHA from 30.5% to 54.8% (Yue et al. 2025). TLL immobilized on APTES–glutaraldehyde-modified magnetic nanoparticles, achieved up to a 4.4-fold increase in DHA content (Matuoog et al. 2018). Structured lipid synthesis approaches also demonstrated high incorporation efficiencies, with CALB on meso-MIL-88 A(Fe) yielding phosphatidyl-EPA and phosphatidyl-DHA levels around 32–35% (Li et al. 2025). Additionally, immobilized *C. rugosa* lipase on hierarchical mesoporous silica increased total ω-3 PUFA content from 32.24% to 53.42% (Dong et al. 2024). Collectively, the literature demonstrates that carefully selected immobilization strategies can substantially enhance EPA and/or DHA enrichment, with different systems exhibiting distinct selectivity profiles depending on enzyme characteristics and support architectures.

**Table 1:** Performances of different immobilized lipase preparations for the enrichment of EPA and DHA

Lipase (Organism)	Support Material	Reaction Conditions	EPA/DHA	Ref.
*Rhizomucor miehei*	Cross-linked enzymes	40 °C, pH 7.0, 24 h; oil/buffer 1:10	3.9-fold EPA selectivity	(Ahrari et al. 2022)
*Burkholderia cepacia* (BCL)	LX201A resin, glutaraldehyde crosslinking	45 °C, 15 h; oil/buffer 1:1.5; 7.6 mg enzyme per 100 g oil	EPA increased by 33.2%, DHA by 0.78%	(Liu et al. 2024)
*Thermomyces lanuginosus* + *Candida antarctica*	Activated chitosan carriers (co-immobilized)	60 °C, 18 h; TAG-FO/EE mixture 1:2; 2% enzyme (w/w)	EPA + DHA increased from 30.5% to 54.8%	(Yue et al. 2025)
*Bovine pancreatic*	N-succinyl chitosan beads	45 °C, 12 h; oil/water 1:4; 0.8 g enzyme per g oil	EPA + DHA increased from 16.3% to 27.0%	(Cui et al. 2017)
*Candida antarctica* (CALB)	Meso-MIL-88 A(Fe) MOF	50 °C, 24 h; PC: EPA-EE or PC: DHA-EE 1:5; 10% enzyme	Phosphatidyl-EPA ~ 32–33%; phosphatidyl-DHA ~ 35%	(Li et al. 2025)
*Thermomyces lanuginosus* + *Fusarium oxysporum*	Epoxy-polymerized melamine sponge + PEI	55 ℃, 18 h, continuous microflow reactor	DHA/EPA incorporation 54.3%	(Zhou et al. 2024)
*Thermomyces lanuginosus* (TLL)	GMNPs (APTES- and glutaraldehyde-modified Fe_3_O_4_)	40 °C, 14 h, 2 g fish oil with 50% water, 200 mg immobilized enzyme, optimal loading 4.4 mg TLL/g support	DHA increased to 31% (4.4-fold enrichment)	(Matuoog et al. 2018)
*Candida rugosa* (CRL)	Hierarchical porous hollow silica microsphere modified with trimethoxy(propyl)silane (HPHSM-C3)	37 °C, 4 h, tuna oil: PBS = 1:1 (w/w), 12.5 U immobilized enzyme	ω-3 PUFAs in glycerides increased from 32.24% to 53.42% (48.78% hydrolysis);	(Dong et al. 2024)
*Bacillus subtilis* (BSL)	Magnetic nanoparticles (zinc ferrite, covalently immobilized via glutaraldehyde)	Hydrolysis at pH 8.5, 65 °C using 2 U immobilized lipase for 10 min	EPA increased by 18.7%, whereas DHA decreased by 4.8% after immobilization.	(Verma et al. 2019)
*Rhizomucor miehei*	MWCNT/Gen@RML MWCNT/Glu@RML MWCNT/Gly@RML	50 °C, 12–60 h; fish oil/n-hexane/buffer system; pH 7.5; 1–7.5 U lipase	EPA + DHA enrichment: 92.85% (Gen), 82.33% (Glu), 78.58% (Gly)	This study

**Fig. 7:**
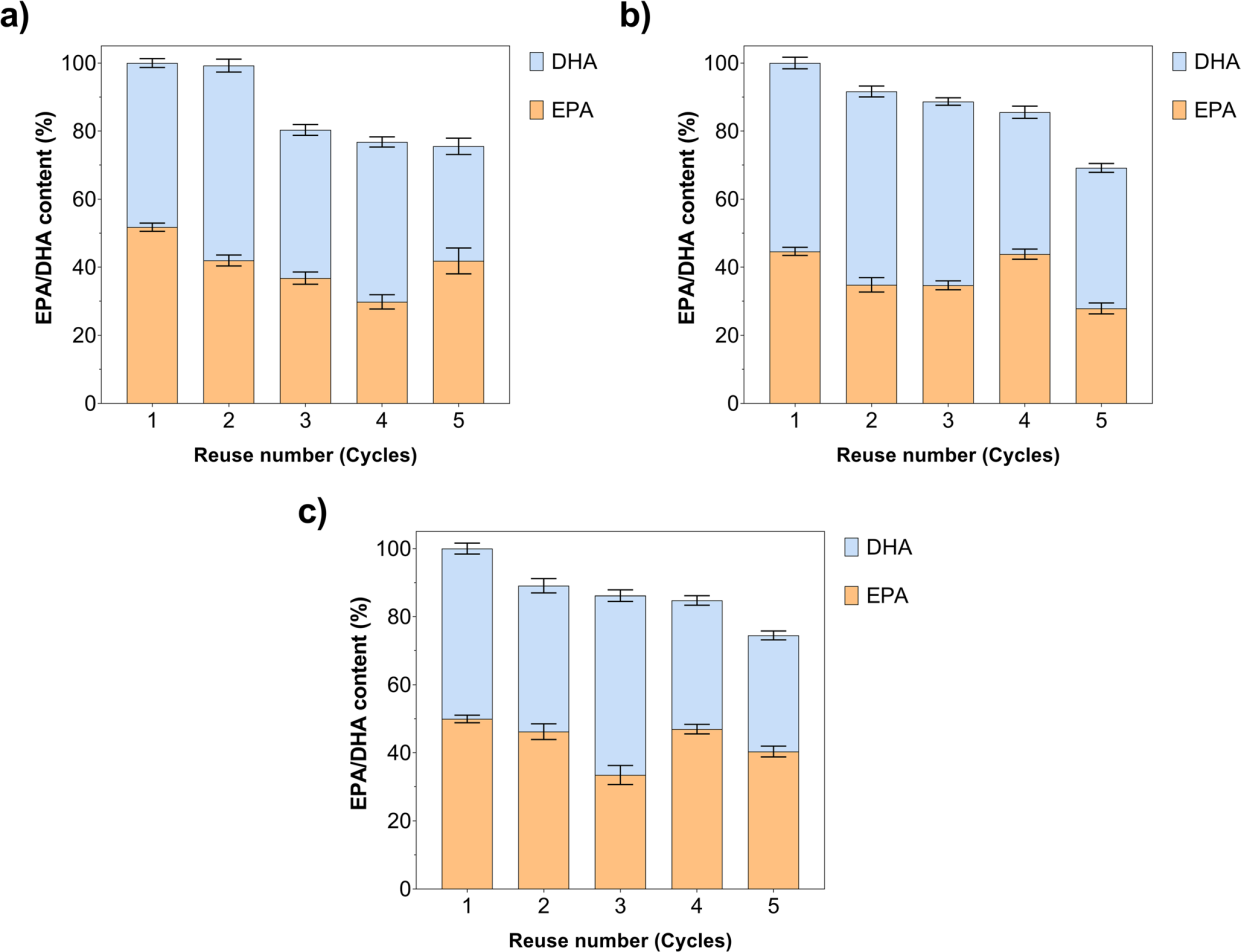
Reusability performance of immobilized RML during enzymatic hydrolysis of fish oil. (**a**) MWCNT/Gen@RML, (**b**) MWCNT/Glu@RML, and (**c**) MWCNT/Gly@RML. The relative EPA and DHA contents (%) were determined over five consecutive reuse cycles using 1 U enzyme at 50 °C and 250 rpm for 24 h under the same reaction conditions. Error bars indicate SD

Reusability experiments demonstrated that MWCNT/Gen@RML, MWCNT/Glu@RML, and MWCNT/Gly@RML retained varying levels of EPA and DHA production over five consecutive cycles (Fig. 7). MWCNT/Gen@RML exhibited the most stable catalytic performance, with EPA content decreasing from 51.8% to 41.9% and DHA from 48.2% to 33.7%, indicating only a moderate loss in activity after repeated use. MWCNT/Gly@RML exhibited a more pronounced decline: EPA decreased from 44.6% to 27.9% and DHA decreased from 55.3% to 41.3%, suggesting partial deactivation over successive cycles. MWCNT/Glu@RML displayed relatively high operational stability, maintaining between 49.9% and 40.4% and showing a gradual decrease in DHA from 50.0% to 34.1%. Considering the overall EPA + DHA yield after five cycles, MWCNT/Gen@RML preserved 75% of its initial total product formation (25% loss); MWCNT/Glu@RML retained 72% (28% loss); and MWCNT/Gly@RML retained 66% (34% loss). Collectively, these findings indicate that immobilization significantly enhanced the operational stability and reusability of RML. Among the immobilized systems, MWCNT/Gen@RML exhibited the highest performance, followed by MWCNT/Glu@RML and MWCNT/Gly@RML, showing a consistent trend in both EPA/DHA productivity and total yield retention across multiple reaction cycles.Ahrari et al. (2024) immobilized *Rhizopus oryzae* lipase (ROL) on octyl-Sepharose and Q Sepharose by adsorption, and on MANAE by covalent immobilization. Both adsorbed ROLs remained at approximately 50% of their initial activity after the third cycle of reuse. However, the covalently immobilized ROL retained 60% of its original activity even after five reuse cycles.Liu et al. (2024) showed that EPA content decreased from 73.8% to 70.6%, while DHA content remained unchanged after 10 consecutive batches when *B. cepacia* lipase immobilized on LX201A resin by glutaraldehyde crosslinking was used.Mohammadi et al. (2015) reported that RML immobilized on epoxy functionalized silica particles (silica-epoxy-RML) and its iminodiacetic acid modified counterpart (silica-epoxy-IDA-RML) retained 83% and 85% of their activities, respectively, after five cycles of the reaction.

## Conclusion

In this study, immobilization of RML on chemically functionalized multi-walled carbon nanotubes through genipin, glutaraldehyde, and glyoxyl coupling chemistries generated stable and efficient biocatalysts for the selective enrichment of ω-3 fatty acids from fish oil. Although all immobilization strategies improved the thermal and operational stability of the enzyme, spacer arm chemistry strongly influenced catalytic behavior and product selectivity. The glutaraldehyde-linked derivative exhibited the highest catalytic efficiency and produced the greatest final EPA concentration. In contrast, the genipin-based system showed faster accumulation of ω-3 fatty acids and achieved the highest enrichment level, reaching 92.85% EPA + DHA enrichment, while also demonstrating superior operational stability by retaining approximately 75% of its initial activity after five reaction cycles. The glyoxyl-based derivative displayed intermediate catalytic activity but showed enhanced tolerance to elevated temperatures. The results further indicate that immobilization chemistry influences product distribution during hydrolysis. While MWCNT/Gen@RML and MWCNT/Gly@RML favored DHA formation, the MWCNT/Glu@RML system promoted EPA enrichment, indicating that spacer arm chemistry can modulate the ω-3 fatty acid profile obtained from lipase-catalyzed hydrolysis of fish oil. Certain limitations of the present study should be acknowledged. In particular, detailed structural characterization of the carrier materials, including pore structure analysis and enzyme localization on the support surface, was not investigated. Such analyses could provide further insight into mass transfer effects and immobilization mechanisms. Future studies should therefore focus on advanced structural characterization of the immobilized systems and on optimizing reaction parameters to further improve catalytic efficiency and selectivity. Evaluation of the long-term stability of these biocatalysts under continuous or larger-scale processing conditions will also be important for potential industrial implementation. These findings demonstrate that rational selection of spacer arm chemistry provides an effective strategy for tuning the catalytic performance and selectivity of immobilized lipases, enabling controlled production of tailored ω-3 fatty acid compositions for nutritional and industrial applications.

## Electronic Supplementary Material

Below is the link to the electronic supplementary material.

Supplementary Material 1

## Data Availability

No datasets were generated or analysed during the current study.

## References

[CR1] Abdelaziz AA, Abo-Kamar AM, Elkotb ES, Al-Madboly LA (2025) Microbial lipases: advances in production, purification, biochemical characterization, and multifaceted applications in industry and medicine. Microb Cell Fact 24:1–20. 10.1186/S12934-025-02664-6 39939876 10.1186/s12934-025-02664-6 PMC11823137

[CR2] Abdul Manan FM, Attan N, Widodo N et al (2018) *Rhizomucor miehei* lipase immobilized on reinforced chitosan–chitin nanowhiskers support for synthesis of eugenyl benzoate. Prep Biochem Biotechnol 48:92–102. 10.1080/10826068.2017.1405021 29194017 10.1080/10826068.2017.1405021

[CR3] Ahmed R, ul ain Hira N, Wang M et al (2024) Genipin, a natural blue colorant precursor: Source, extraction, properties, and applications. Food Chem 434:137498. 10.1016/j.foodchem.2023.137498 37741231 10.1016/j.foodchem.2023.137498

[CR5] Ahrari F, Yousefi M, Habibi Z, Mohammadi M (2022) Application of undecanedicarboxylic acid to prepare cross-linked enzymes (CLEs) of *Rhizomucor miehei* lipase (RML); Selective enrichment of polyunsaturated fatty acids. Mol Catal 520:112172. 10.1016/j.mcat.2022.112172

[CR4] Ahrari F, Pourmohammadi Lish M, Yousefi M, Mohammadi M (2024) Improving the stability of an unstable lipase by applying different immobilization strategies for the selective hydrolysis of fish oil. J Am Oil Chem Soc 101:839–850. 10.1002/aocs.12833

[CR6] Akanbi TO, Adcock JL, Barrow CJ (2013) Selective concentration of EPA and DHA using *Thermomyces lanuginosus* lipase is due to fatty acid selectivity and not regioselectivity. Food Chem 138:615–620. 10.1016/j.foodchem.2012.11.007 23265531 10.1016/j.foodchem.2012.11.007

[CR7] Akbulut K, Taranacı S, Özkök S et al (2025) Heterologous expression of calcium-independent mesophilic α-amylase from *Priestia megaterium*: Immobilization on genipin-modified multi-walled carbon nanotubes and silica supports to enhance thermostability and catalytic activity. Bioorg Chem 155:108151. 10.1016/j.bioorg.2025.108151 39799729 10.1016/j.bioorg.2025.108151

[CR8] Alagöz D, Toprak A, Yildirim D et al (2021) Modified silicates and carbon nanotubes for immobilization of lipase from *Rhizomucor miehei*: Effect of support and immobilization technique on the catalytic performance of the immobilized biocatalysts. Enzyme Microb Technol 144:109739. 10.1016/j.enzmictec.2020.109739 33541574 10.1016/j.enzmictec.2020.109739

[CR9] Alijani S, Hahn A, Harris WS, Schuchardt JP (2025) Bioavailability of EPA and DHA in humans – A comprehensive review. Prog Lipid Res 97:101318. 10.1016/j.plipres.2024.101318 39736417 10.1016/j.plipres.2024.101318

[CR10] Almeida MR, Cristóvão RO, Barros MA et al (2021) Superior operational stability of immobilized l-asparaginase over surface-modified carbon nanotubes. Sci Rep 11:21529. 10.1038/s41598-021-00841-2 34728685 10.1038/s41598-021-00841-2 PMC8563809

[CR11] Asmat S, Anwer AH, Husain Q (2019) Immobilization of lipase onto novel constructed polydopamine grafted multiwalled carbon nanotube impregnated with magnetic cobalt and its application in synthesis of fruit flavours. Int J Biol Macromol 140:484–495. 10.1016/j.ijbiomac.2019.08.086 31408654 10.1016/j.ijbiomac.2019.08.086

[CR12] Baena A, Orjuela A, Rakshit SK, Clark JH (2022) Enzymatic hydrolysis of waste fats, oils and greases (FOGs): Status, prospective, and process intensification alternatives. Chem Eng Process 175:108930. 10.1016/j.cep.2022.108930

[CR13] Barbosa O, Ortiz C, Berenguer-Murcia Á et al (2013) Glutaraldehyde in bio-catalysts design: a useful crosslinker and a versatile tool in enzyme immobilization. RSC Adv 4:1583–1600. 10.1039/c3ra45991h

[CR14] Betancor L, López-Gallego F, Hidalgo A et al (2006) Different mechanisms of protein immobilization on glutaraldehyde activated supports: Effect of support activation and immobilization conditions. Enzyme Microb Technol 39:877–882. 10.1016/j.enzmictec.2006.01.014

[CR15] Bilal M, Degorska O, Szada D et al (2024) Support Materials of Organic and inorganic origin as platforms for horseradish peroxidase immobilization: Comparison study for high stability and activity recovery. Molecules 29:710. 10.3390/molecules29030710 38338454 10.3390/molecules29030710 PMC10856027

[CR16] Boratyński J, Zal T (1990) Colorimetric micromethods for glutaraldehyde determination by means of phenol and sulfuric acid or phenol and perchloric acid. Anal Biochem 184:259–262. 10.1016/0003-2697(90)90677-2 2158247 10.1016/0003-2697(90)90677-2

[CR17] Boudrant J, Woodley JM, Fernandez-Lafuente R (2020) Parameters necessary to define an immobilized enzyme preparation. Process Biochem 90:66–80. 10.1016/j.procbio.2019.11.026

[CR18] Castejón N, Señoráns FJ (2020) Enzymatic modification to produce health-promoting lipids from fish oil, algae and other new omega-3 sources: A review. N Biotechnol 57:45–54. 10.1016/j.nbt.2020.02.006 32224214 10.1016/j.nbt.2020.02.006

[CR19] Chandra K, Dong C, Di, Chauhan AS et al (2025) Advancements in lipase immobilization: Enhancing enzyme efficiency with nanomaterials for industrial applications. Int J Biol Macromol 311:143754. 10.1016/j.ijbiomac.2025.143754 40318715 10.1016/j.ijbiomac.2025.143754

[CR20] Cowan DA, Fernandez-Lafuente R (2011) Enhancing the functional properties of thermophilic enzymes by chemical modification and immobilization. Enzyme Microb Technol 49:326–346. 10.1016/j.enzmictec.2011.06.023 22112558 10.1016/j.enzmictec.2011.06.023

[CR21] Cui S, Zhou QW, Wang XL et al (2017) Immobilization of lipase onto N-succinyl-chitosan beads and its application in the enrichment of polyunsaturated fatty acids in fish oil. J Food Biochem 41:e12395. 10.1111/jfbc.12395

[CR22] Dong Z, Jin J, Wei W et al (2024) Fabrication of immobilized lipases from Candida rugosa on hierarchical mesoporous silica for enzymatic enrichment of ω-3 polyunsaturated fatty acids by selective hydrolysis. Food Chem X 22:101434. 10.1016/j.fochx.2024.101434 38779499 10.1016/j.fochx.2024.101434 PMC11108833

[CR23] Du JR, Hsu LH, Xiao ES et al (2020) Using genipin as a green crosslinker to fabricate chitosan membranes for pervaporative dehydration of isopropanol. Sep Purif Technol 244:116843. 10.1016/j.seppur.2020.116843

[CR24] Dutra Rosolen M, Gennari A, Volpato G, de Souza CFV (2017) Biocatalytic characterization of *Aspergillus oryzae* β-galactosidase immobilized on functionalized multi-walled carbon nanotubes. Biocatal Biotransform 35:260–268. 10.1080/10242422.2017.1323886

[CR25] Esparza-Flores EE, Cardoso FD, Siquiera LB et al (2023) Genipin crosslinked porous chitosan beads as robust supports for β-galactosidase immobilization: Characterization, stability, and bioprocessing potential. Int J Biol Macromol 250:126234. 10.1016/j.ijbiomac.2023.126234 37567531 10.1016/j.ijbiomac.2023.126234

[CR26] Fan Y, Su F, Li K et al (2017) Carbon nanotube filled with magnetic iron oxide and modified with polyamidoamine dendrimers for immobilizing lipase toward application in biodiesel production. Sci Rep 7:45643. 10.1038/srep45643 28358395 10.1038/srep45643 PMC5372472

[CR27] Ghide MK, Li K, Wang J et al (2022) Immobilization of *Rhizomucor miehei* lipase on magnetic multiwalled carbon nanotubes towards the synthesis of structured lipids rich in sn-2 palmitic acid and sn-1,3 oleic acid (OPO) for infant formula use. Food Chem 390:133171. 10.1016/j.foodchem.2022.133171 35551020 10.1016/j.foodchem.2022.133171

[CR28] Gonzalez-Soto M, Mutch DM (2021) Diet Regulation of long-chain PUFA synthesis: Role of macronutrients, micronutrients, and polyphenols on ∆-5/∆-6 desaturases and elongases 2/5. Adv Nutr 12:980–994. 10.1093/advances/nmaa142 33186986 10.1093/advances/nmaa142 PMC8166571

[CR29] Grmasha RA, Al-sareji OJ, Meiczinger M et al (2024) A sustainable nano-hybrid system of laccase@M-MWCNTs for multifunctional PAHs and PhACs removal from water, wastewater, and lake water. Environ Res 246:118097. 10.1016/j.envres.2024.118097 38176629 10.1016/j.envres.2024.118097

[CR30] Guimarães JR, Oliveira KSGC, Gonçalves MCP et al (2023) A review of lipase immobilization on hydrophobic supports incorporating systematic mapping principles. React Chem Eng 8:2689–2702. 10.1039/d3re00420a

[CR31] Guisán JM (1988) Aldehyde-agarose gels as activated supports for immobilization-stabilization of enzymes. Enzyme Microb Technol 10:375–382. 10.1016/0141-0229(88)90018-x

[CR32] Gupta N, Rathi P, Gupta R (2002) Simplified para-nitrophenyl palmitate assay for lipases and esterases. Anal Biochem 311:98–99. 10.1016/S0003-2697(02)00379-2 12441161 10.1016/s0003-2697(02)00379-2

[CR33] Hughes KJ, Iyer KA, Bird RE et al (2024) Review of carbon nanotube research and development: materials and emerging applications. ACS Appl Nano Mater 7:18695–18713. 10.1021/acsanm.4c02721

[CR34] Ismail AR, Baek KH (2020) Lipase immobilization with support materials, preparation techniques, and applications: Present and future aspects. Int J Biol Macromol 163:1624–1639. 10.1016/j.ijbiomac.2020.09.021 32916199 10.1016/j.ijbiomac.2020.09.021

[CR35] Jun LY, Mubarak NM, Yon LS et al (2019) Immobilization of peroxidase on functionalized MWCNTs-buckypaper/polyvinyl alcohol nanocomposite membrane. Sci Rep 9:2215. 10.1038/s41598-019-39621-4 30778111 10.1038/s41598-019-39621-4 PMC6379398

[CR36] Kar A, Ghosh P, Patra P et al (2023) Omega-3 fatty acids mediated Cellular signaling and its regulation in Human Health. Clin Nutr Open Sci 52:72–86. 10.1016/j.nutos.2023.10.004

[CR37] Karia M, Kaspal M, Alhattab M, Puri M (2024) Marine-derived lipases for enhancing enrichment of very-long-chain polyunsaturated fatty acids with reference to omega-3 fatty acids. Mar Drugs 22:301. 10.3390/md22070301 39057410 10.3390/md22070301 PMC11277628

[CR38] Kosti RI, Kasdagli MI, Kyrozis A et al (2022) Fish intake, n-3 fatty acid body status, and risk of cognitive decline: a systematic review and a dose-response meta-analysis of observational and experimental studies. Nutr Rev 80:1445–1458. 10.1093/nutrit/nuab078 34605891 10.1093/nutrit/nuab078

[CR39] Kotsoni E, Daukšas E, Aas GH et al (2024) Quality assessment of fish oil obtained after enzymatic hydrolysis of a mixture of rainbow trout (*Oncorhynchus mykiss*) and Atlantic salmon (*Salmo salar*) rest raw material pretreated by high pressure. Mar Drugs 22:261. 10.3390/md22060261 38921572 10.3390/md22060261 PMC11205082

[CR40] Li Y, Wu G, Liu Z et al (2025) Enzymatic synthesis of phosphatidyl-EPA/DHA using *Candida antarctica* lipase B immobilized on mesoporous MIL-88 A. Bioresour Bioprocess 12:123. 10.1186/s40643-025-00959-5 41182440 10.1186/s40643-025-00959-5 PMC12583253

[CR41] Liu R, Li P, Li S et al (2024) Immobilized Burkholderia cepacia lipase and its application in selective enrichment of EPA by hydrolysis of fish oil. LWT 206:116598. 10.1016/j.lwt.2024.116598

[CR42] Massoumi B, Mohammad-Rezaei R, Abbasian M, Jaymand M (2019) Amine-functionalized carbon nanotubes as curing agent for polystyrene-modified novolac epoxy resin: synthesis, characterization and possible applications. Appl Phys A 125:304. 10.1007/s00339-019-2599-4

[CR43] Mateo C, Abian O, Bernedo M et al (2005) Some special features of glyoxyl supports to immobilize proteins. Enzyme Microb Technol 37:456–462. 10.1016/j.enzmictec.2005.03.020

[CR44] Mateo C, Palomo JM, Fuentes M et al (2006) Glyoxyl agarose: A fully inert and hydrophilic support for immobilization and high stabilization of proteins. Enzyme Microb Technol 39:274–280. 10.1016/j.enzmictec.2005.10.014

[CR45] Matuoog N, Li K, Yan Y (2018) *Thermomyces lanuginosus* lipase immobilized on magnetic nanoparticles and its application in the hydrolysis of fish oil. J Food Biochem 42:e12549. 10.1111/jfbc.12549

[CR46] Migneault I, Dartiguenave C, Bertrand MJ, Waldron KC (2004) Glutaraldehyde: Behavior in aqueous solution, reaction with proteins, and application to enzyme crosslinking. Biotechniques 37:790–802. 10.2144/04375rv01 15560135 10.2144/04375RV01

[CR47] Miguez JP, Fernandez-Lafuente R, Tavano OL, Mendes AA (2023) The immobilization and stabilization of trypsin from the porcine pancreas on chitosan and its catalytic performance in protein hydrolysis. Catalysts 13:10–1344. 10.3390/catal13101344

[CR48] Moguei MRS, Habibi Z, Shahedi M et al (2022) Immobilization of *Thermomyces lanuginosus* lipase through isocyanide-based multi component reaction on multi-walled carbon nanotube: application for kinetic resolution of rac-ibuprofen. Biotechnol Rep 35:e00759. 10.1016/j.btre.2022.e00759 10.1016/j.btre.2022.e00759 PMC9434027 36060211

[CR49] Mohammadi M, Habibi Z, Dezvarei S et al (2015) Selective enrichment of polyunsaturated fatty acids by hydrolysis of fish oil using immobilized and stabilized *Rhizomucor miehei* lipase preparations. Food Bioprod Process 94:414–421. 10.1016/j.fbp.2014.05.007

[CR50] Monsan P (1978) Optimization of glutaraldehyde activation of a support for enzyme immobilization. J Mol Catal 3:371–384. 10.1016/0304-5102(78)80026-1

[CR51] Moreira KdaS, de Oliveira ALB, de Júnior LS M, et al (2020) Lipase From *Rhizomucor miehei* immobilized on magnetic nanoparticles: Performance in fatty acid ethyl ester (FAEE) optimized production by the Taguchi Method. Front Bioeng Biotechnol 8:544875. 10.3389/FBIOE.2020.00693 10.3389/fbioe.2020.00693 PMC7338345 32695765

[CR52] Okura NS, Sabi GJ, Crivellenti MC et al (2020) Improved immobilization of lipase from *Thermomyces lanuginosus* on a new chitosan-based heterofunctional support: Mixed ion exchange plus hydrophobic interactions. Int J Biol Macromol 163:550–561. 10.1016/j.ijbiomac.2020.07.021 32645498 10.1016/j.ijbiomac.2020.07.021

[CR53] Oliver L, Dietrich T, Marañón I et al (2020) Producing omega-3 polyunsaturated fatty acids: A review of sustainable sources and future trends for the EPA and DHA market. Resources 9:148. 10.3390/resources9120148

[CR54] Ongis M, Liese D, Di Marcoberardino G et al (2025) Modeling of enzymatic transesterification for omega-3 fatty acids enrichment in fish oil. Food Chem 463:141379. 10.1016/j.foodchem.2024.141379 39362151 10.1016/j.foodchem.2024.141379

[CR55] Özdemir Fİ, Karaaslan B, Tülek A et al (2023) Immobilization of recombinant l-asparaginase from *Geobacillus kaustophilus* on magnetic MWCNT-nickel composites. Process Biochem 127:10–20. 10.1016/j.procbio.2023.01.021

[CR56] Özdemir Fİ, Tülek A, Karaaslan B, Yildirim D (2024) Evaluation of multi-walled carbon nanotubes bearing aldehyde groups of different lengths for the immobilization of *Geobacillus kaustophilus* l-asparaginase. Mol Catal 555:113903. 10.1016/j.mcat.2024.113903

[CR57] Pinheiro BB, Saibi S, Haroune L et al (2023) Genipin and glutaraldehyde based laccase two-layers immobilization with improved properties: New biocatalysts with high potential for enzymatic removal of trace organic contaminants. Enzyme Microb Technol 169:110261. 10.1016/j.enzmictec.2023.110261 37269616 10.1016/j.enzmictec.2023.110261

[CR58] Qiao M, Hua X, Yuan Y, Cao X (2024) Enrichment of EPA and DHA by enzymatic ethanolysis: Effects of lipases from Thermomyces lanuginosus, Rhizomucor miehei, Rhizopus oryzae and Candida antarctica. J Am Oil Chem Soc 101:1197–1208. 10.1002/aocs.12800

[CR59] Qin J, Kurt E, LBassi T et al (2023) Biotechnological production of omega-3 fatty acids: current status and future perspectives. Front Microbiol 14:1280296. 10.3389/fmicb.2023.1280296 38029217 10.3389/fmicb.2023.1280296 PMC10662050

[CR60] Rathinavel S, Priyadharshini K, Panda D (2021) A review on carbon nanotube: An overview of synthesis, properties, functionalization, characterization, and the application. Mater Sci Eng B 268:115095. 10.1016/j.mseb.2021.115095

[CR61] Rodrigues RC, Fernandez-Lafuente R (2010) Lipase from Rhizomucor miehei as an industrial biocatalyst in chemical process. J Mol Catal B Enzym 64:1–22. 10.1016/j.molcatb.2010.02.003

[CR62] Sampaio CS, Angelotti JAF, Fernandez-Lafuente R, Hirata DB (2022) Lipase immobilization via cross-linked enzyme aggregates: Problems and prospects – A review. Int J Biol Macromol 215:434–449. 10.1016/j.ijbiomac.2022.06.139 35752332 10.1016/j.ijbiomac.2022.06.139

[CR64] Schmid RD, Verger R (1998) Lipases: Interfacial enzymes with attractive applications. Angew Chem Int Ed 37:1608–1633. 10.1002/(SICI)1521-3773(19980703)37:12<1608::AID-ANIE1608>3.0.CO;2-V 10.1002/(SICI)1521-3773(19980703)37:12<1608::AID-ANIE1608>3.0.CO;2-V 29711530

[CR63] Sheldon RA (2010) Cross-linked enzyme aggregates as industrial biocatalysts. Org Process Res Dev 15:213–223. 10.1021/op100289f

[CR65] Stergiou PY, Foukis A, Filippou M et al (2013) Advances in lipase-catalyzed esterification reactions. Biotechnol Adv 31:1846–1859. 10.1016/j.biotechadv.2013.08.006 23954307 10.1016/j.biotechadv.2013.08.006

[CR66] Sulym I, Zdarta J, Ciesielczyk F et al (2021) Pristine and poly(dimethylsiloxane) modified multi-walled carbon nanotubes as supports for lipase immobilization. Materials 14:2874. 10.3390/ma14112874 34072043 10.3390/ma14112874 PMC8198216

[CR67] Swetha N, Mathanghi SK (2024) Towards sustainable omega-3 fatty acids production – A comprehensive review on extraction methods, oxidative stability and bio-availability enhancement. Food Chem Adv 4:100603. 10.1016/j.focha.2023.100603

[CR68] Tacias-Pascacio VG, Abellanas-Perez P, de Andrades D et al (2025) A comprehensive review of lipase-catalyzed acidolysis as a method for producing structured glycerides. Int J Biol Macromol 309:142878. 10.1016/j.ijbiomac.2025.142878 40194578 10.1016/j.ijbiomac.2025.142878

[CR69] Tülek A (2025) Entrapment of *Geobacillus kaustophilus* lipase in ZIF-8 and cross-linking with genipin for biodiesel production from vegetable oils. Mol Catal 576:114919. 10.1016/j.mcat.2025.114919

[CR70] Tülek A, Günay E, Servili B et al (2023) Sustainable production of formic acid from CO_2_ by a novel immobilized mutant formate dehydrogenase. Sep Purif Technol 309:123090. 10.1016/j.seppur.2022.123090

[CR71] Tülek A, Şükür G, Özdemir Fİ et al (2025) Immobilization of L-asparaginase on genipin and divinyl sulfone cross-linked multi-walled carbon nanotubes and silica supports for acrylamide mitigation. Food Chem 491:145268. 10.1016/j.foodchem.2025.145268 40561764 10.1016/j.foodchem.2025.145268

[CR72] Verger R (1997) Interfacial activation’ of lipases: facts and artifacts. Trends Biotechnol 15:32–38. 10.1016/S0167-7799(96)10064-0

[CR73] Verma ML, Rao NM, Tsuzuki T et al (2019) Suitability of recombinant lipase immobilised on functionalised magnetic nanoparticles for fish oil hydrolysis. Catalysts 9:420. 10.3390/catal9050420

[CR74] Vescovi V, Kopp W, Guisán JM et al (2016) Improved catalytic properties of *Candida antarctica* lipase B multi-attached on tailor-made hydrophobic silica containing octyl and multifunctional amino- glutaraldehyde spacer arms. Process Biochem 51:2055–2066. 10.1016/j.procbio.2016.09.016

[CR75] Wahba MI (2024) A comprehensive review on genipin: an efficient natural cross-linker for biopolymers. Polym Bull 81:14251–14305. 10.1007/S00289-024-05406-7

[CR76] Xu L, Li J, Zhang H et al (2025) Biological modification and industrial applications of microbial lipases: A general review. Int J Biol Macromol 302:140486. 10.1016/j.ijbiomac.2025.140486 39889982 10.1016/j.ijbiomac.2025.140486

[CR77] Yan Q, Li Z, Sun R et al (2025) Promoted expression of a lipase for its application in EPA/DHA enrichment and mechanistic insights into its substrate specificity. Int J Biol Macromol 296:139628. 10.1016/j.ijbiomac.2025.139628 39798747 10.1016/j.ijbiomac.2025.139628

[CR78] Yi M, You Y, Zhang Y et al (2023) Highly Valuable fish oil: Formation process, enrichment, subsequent utilization, and storage of eicosapentaenoic acid ethyl esters. Molecules 28:672. 10.3390/molecules28020672 36677730 10.3390/molecules28020672 PMC9865908

[CR79] Yousefi M, Marciello M, Guisan JM et al (2020) Fine modulation of the catalytic properties of *Rhizomucor miehei* lipase driven by different immobilization strategies for the selective hydrolysis of fish oil. Molecules 25:545. 10.3390/molecules25030545 32012738 10.3390/molecules25030545 PMC7037125

[CR81] Yu J, Shen C, Chen H et al (2024) Lipase-catalyzed preparation, bioavailability and functional properties of a DHA-enriched tuna oil. LWT 203:116341. 10.1016/j.lwt.2024.116341

[CR80] Yu J, Fu Y, Tang X et al (2025) Enrichment of EPA and DHA in glycerides by selective enzymatic ethanolysis. Food Chem 463:141226. 10.1016/j.foodchem.2024.141226 39270490 10.1016/j.foodchem.2024.141226

[CR82] Yuan Y, Shen J, Salmon S (2023) Developing enzyme immobilization with fibrous membranes: Longevity and characterization considerations. Membr (Basel) 13:532. 10.3390/membranes13050532 10.3390/membranes13050532 PMC10221158 37233593

[CR83] Yue G, Cai Z, Meng C, Mao Y (2025) Synergistic enhancement of lipase catalysis via co-immobilization and chitosan-assisted crosslinking: a streamlined approach for EPA/DHA enrichment in fish oil. Bioprocess Biosyst Eng 2025:1–18. 10.1007/s00449-025-03246-x 10.1007/s00449-025-03246-x 41171416

[CR84] Zhang H, Liu T, Zhu Y et al (2020) Lipases immobilized on the modified polyporous magnetic cellulose support as an efficient and recyclable catalyst for biodiesel production from Yellow horn seed oil. Renew Energy 145:1246–1254. 10.1016/j.renene.2019.06.031

[CR85] Zhou Q, Long NB, Zhang RF (2024) Efficient synthesis of DHA/EPA-rich phosphatidylcholine using immobilized phospholipase A1 on a novel microflow support. Biochem Eng J 207:109296. 10.1016/j.bej.2024.109296

