# Supplementary material for: Tailoring ω-3 fatty acid enrichment through genipin, glutaraldehyde, and glyoxyl linked immobilization of Rhizomucor miehei lipase on MWCNTs
Source: World J Microbiol Biotechnol. 2026 May 12;42(6):282. doi: 10.1007/s11274-026-05011-y (PMC13167907; doi:10.1007/s11274-026-05011-y)
Supplement: Supplementary file 1 — Supplementary Material 1 [file 11274_2026_5011_MOESM1_ESM.docx]

**Supplementary Files**

**Tailoring ω-3 fatty acid enrichment through genipin, glutaraldehyde, and glyoxyl linked immobilization of *Rhizomucor miehei* lipase on MWCNTs**

^a^ Cukurova University, Faculty of Engineering, Department of Chemical Engineering, 01330, Adana/Türkiye

^b^ Cukurova University, Faculty of Science and Letters, Department of Chemistry, 01330, Adana/Türkiye

ORCID: 0000-0002-5041-8160, e-mail: [dyildirim@cu.edu.tr](mailto:dyildirim@cu.edu.tr)

^c^ Kütahya Health Sciences University, Faculty of Engineering and Natural Sciences, Department of Molecular Biology and Genetics, Merkez, 43100, Kütahya/Türkiye, ORCID: 0000-0003-1079-7837, e-mail: [ahmet.tulek@ksbu.edu.tr](mailto:ahmet.tulek@ksbu.edu.tr)

^d^ Iğdır University, Postgraduate Education Institute, Department of Biology, 76000, Iğdır/Türkiye, ORCID: 0009-0004-6491-0406, e-mail: [nurettin.pacal@igdir.edu.tr](mailto:nurettin.pacal@igdir.edu.tr)

^b^ Cukurova University, Faculty of Science and Letters, Department of Chemistry, 01330, Adana/Türkiye, ORCID: 0000-0003-1570-9509, e-mail: [evaran@cu.edu.tr](mailto:evaran@cu.edu.tr)

^e^ Nevsehir Haci Bektas Veli University, Acigol Vocational School, Acigol, 50140 Nevsehir/Türkiye, ORCID: 0000-0002-6520-3226, e-mail: [alitoprak@nevsehir.edu.tr](mailto:alitoprak@nevsehir.edu.tr)

^f^ Cukurova University, Imamoglu Vocational School, Imamoglu, 01700, Adana/Türkiye, ORCID: 0000-0001-6761-6385, e-mail: [dalagoz@cu.edu.tr](mailto:dalagoz@cu.edu.tr)

^b^ Cukurova University, Faculty of Science and Letters, Department of Chemistry, 01330, Adana/Türkiye, ORCID: 0000-0002-3592-8824, e-mail: [rbilgin@cu.edu.tr](mailto:rbilgin@cu.edu.tr)

**Fig. S1.** Immobilization scheme of on MWCNT/Gen (a), MWCNT/Glu (b) and MWCNT/Gly (c).


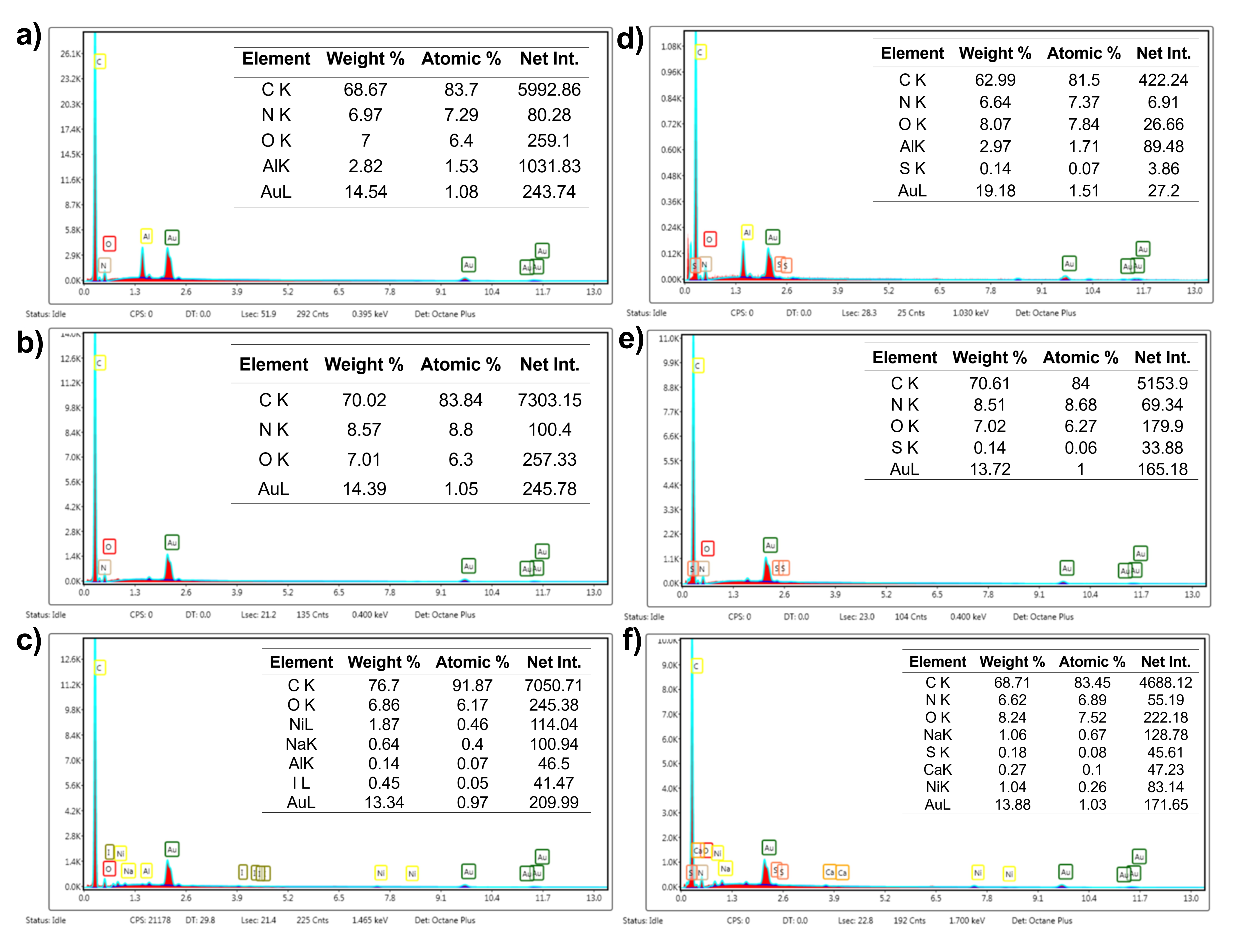


**Fig. S2.** SEM-EDS spectra of the modified MWCNT support materials before and after RML immobilization. Images a–c illustrates the elemental profiles of MWCNT/Gen, MWCNT/Glu and MWCNT/Gly(Ald) prior to enzyme loading, whereas images d–f display the corresponding spectra following RML immobilization, reflecting compositional changes associated with enzyme attachment.


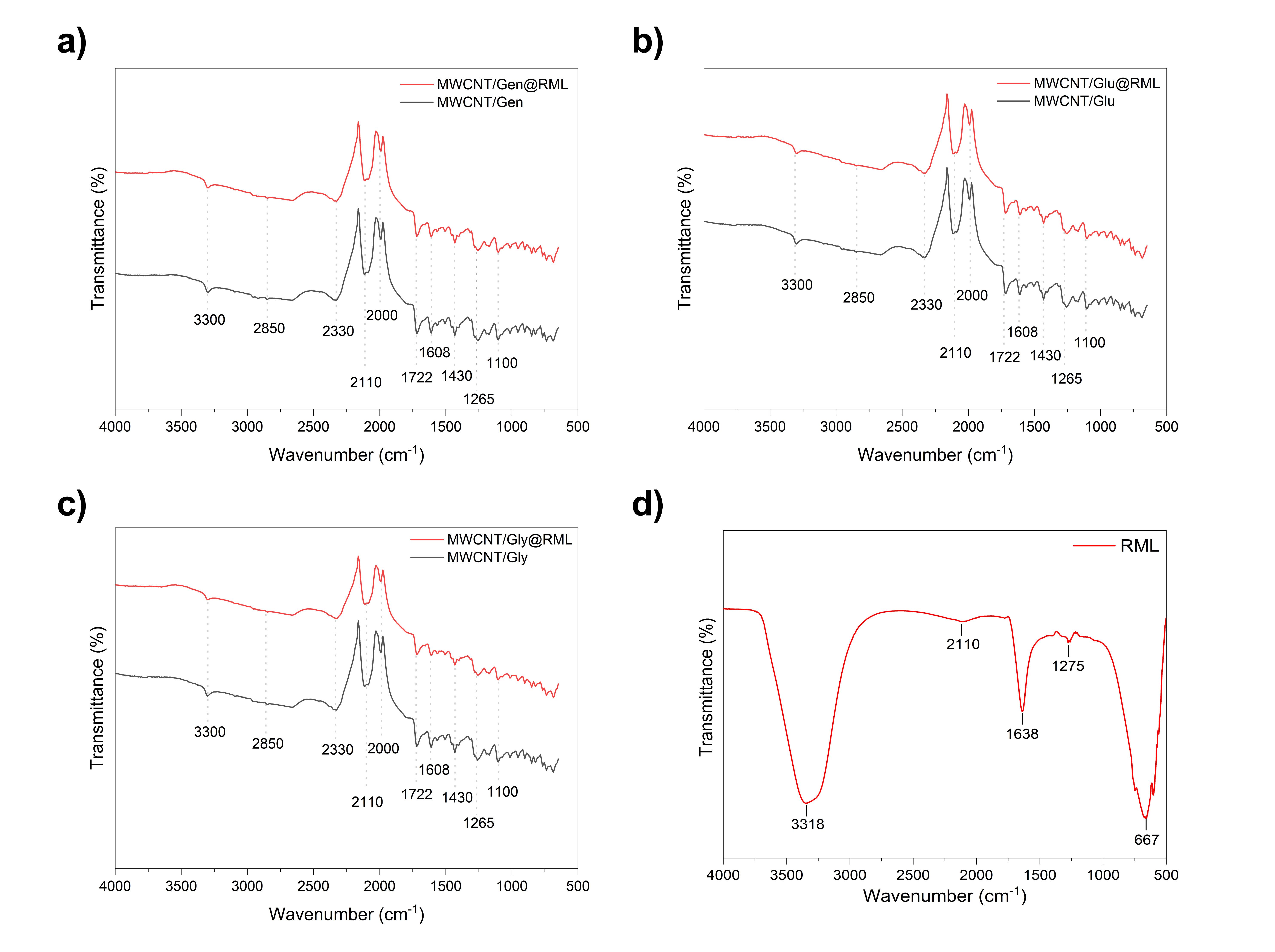


**Fig. S3.** FTIR spectra of (a) MWCNT/Gen and MWCNT/Gen@RML, (b) MWCNT/Glu and MWCNT/Glu@RML, (c) MWCNT/Gly and MWCNT/Gly@RML, and (d) free RML at 500-4000 cm⁻^1^.


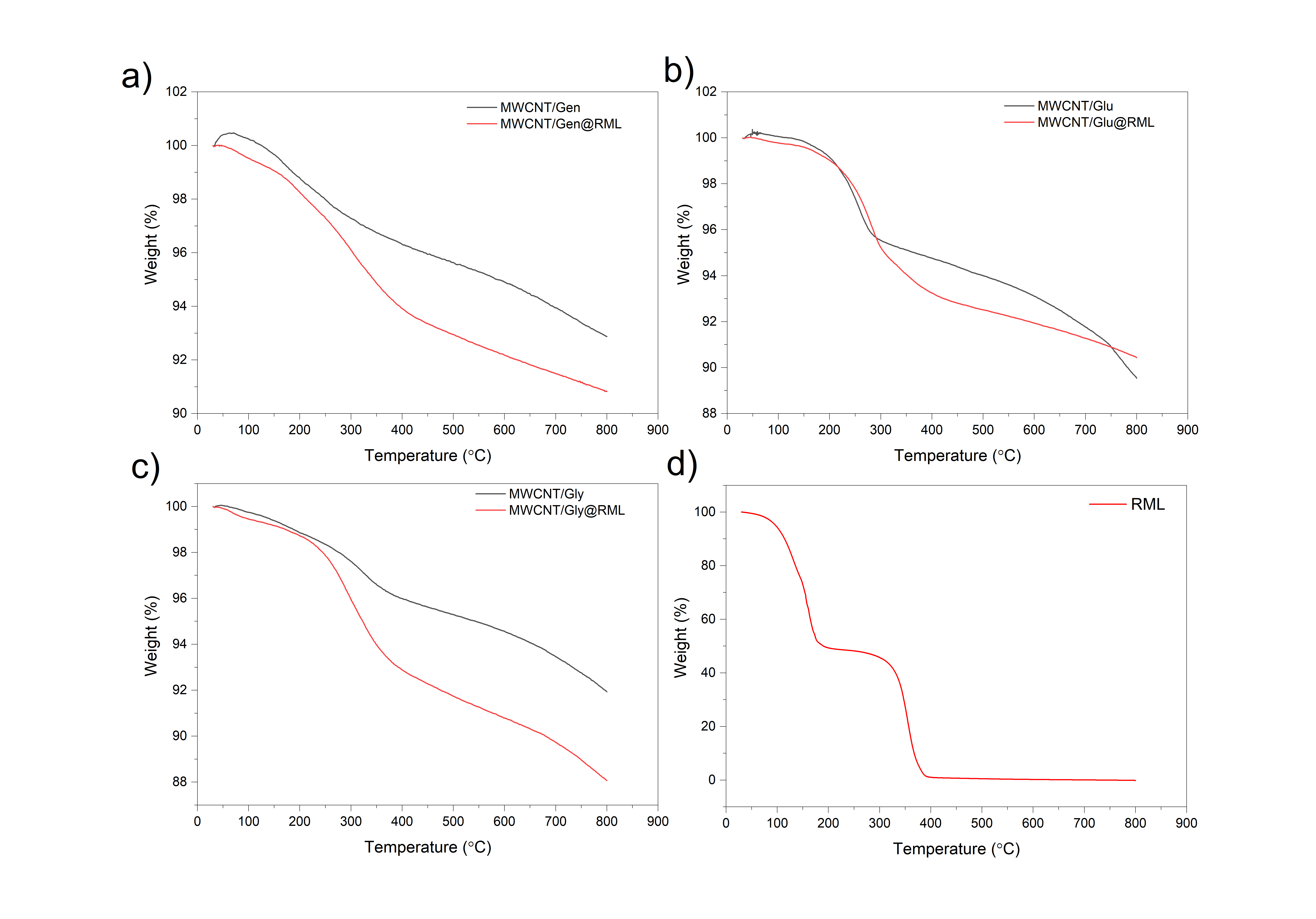


**Fig. S4.** TGA curves showing the thermal decomposition profiles of MWCNT based materials before and after immobilization of Rhizomucor miehei lipase (RML). (a) MWCNT/Gen and MWCNT/Gen@RML, (b) MWCNT/Glu and MWCNT/Glu@RML, (c) MWCNT/Gly and MWCNT/Gly@RML, and (d) free RML. The thermograms illustrate the weight loss behavior of the functionalized MWCNT materials and the corresponding enzyme immobilized biocatalysts, highlighting the additional organic content associated with enzyme loading. Measurements were performed from 25 °C to 800 °C.


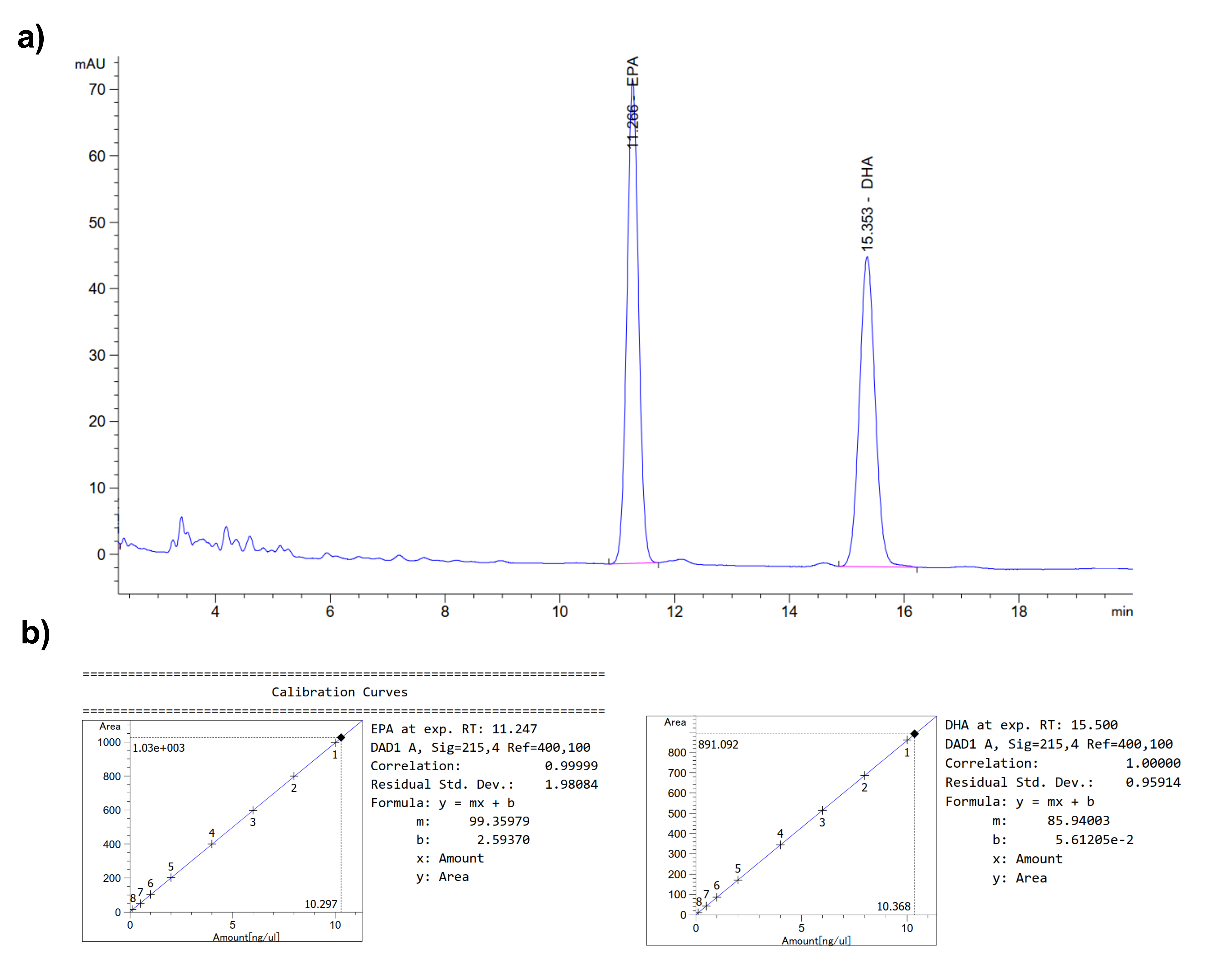


**Fig. S5.** HPLC chromatogram of EPA and DHA standards and their corresponding calibration curves. (a) Representative chromatogram showing EPA (RT = 11.25 min) and DHA (RT = 15.53 min). (b) Calibration curves obtained from EPA and DHA standard solutions.


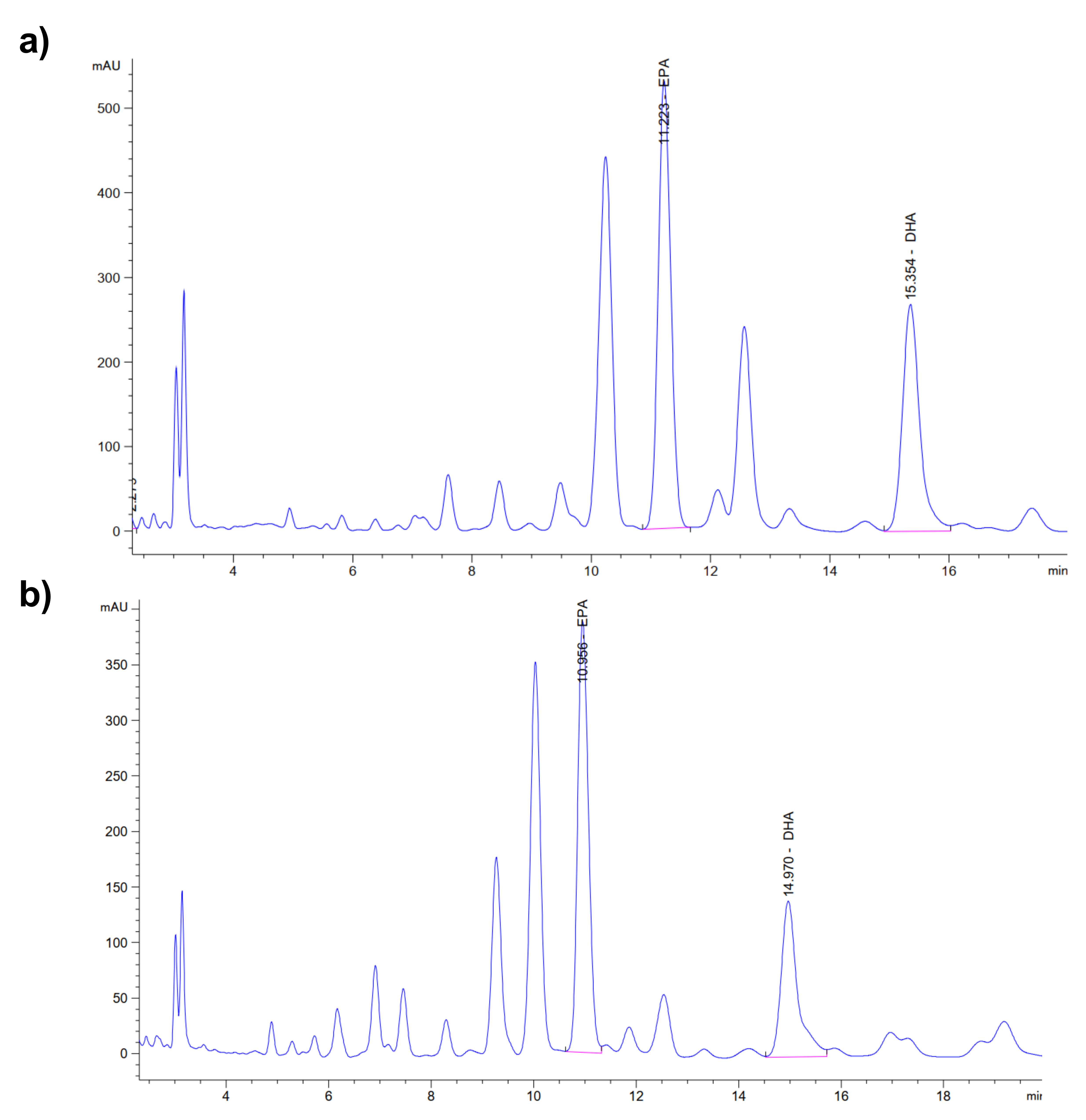


**Fig. S6.** Representative HPLC chromatograms of post-catalytic reaction mixtures obtained after lipase-catalyzed hydrolysis of fish oil. (a–b) Typical chromatograms showing EPA and DHA peaks identified based on their retention times determined using standard solutions.

**Table S1.** Immobilization yield and expressed activity values of RML immobilized on MWCNT via different spacer arms at different immobilization pH values. Values are presented as mean ± SD (n = 3).

| **Enzymes** | **Immobilization**  **pH** | **Immobilization**  **yield (%)** | **Expressed**  **activity (%)** |
| --- | --- | --- | --- |
| MWCNT/Gen@RML | 5.0 | 84.4±1.3 | 59.5±2.2 |
|  | 7.0 | 84.0±1.6 | 38.9±1.5 |
|  | 10.0 | 78.7±2.1 | 25.5±1.6 |
| MWCNT/Glu@RML | 5.0 | 80.4±1.8 | 28.6±1.1 |
|  | 7.0 | 81.1±2.1 | 42.9±2.1 |
|  | 10.0 | 73.1±1.4 | 30.0±1.4 |
| MWCNT/Gly@RML | 5.0 | 80.8±1.8  80.4±2.0  86.1±1.6 | 25.9±0.9  27.2±1.3  38.4±1.6 |
|  | 7.0 |  |  |
|  | 10.0 |  |  |

**Table S2.** Thermal stability parameters of free and immobilized RML at 55°C and 60 °C.

| **Enzyme** | **k_d_ (h⁻¹)** | |  | **t_1/2_ (h)** | |  | **SF** | |
| --- | --- | --- | --- | --- | --- | --- | --- | --- |
|  | **55 °C** | **60 °C** |  | **55 °C** | **60 °C** |  | **55 °C** | **60 °C** |
| free RML | 0.120 | 0.320 |  | 5.7 | 2.1 |  | 1.0 | 1.0 |
| MWCNT/Gen@RML | 0.0101 | 0.0094 |  | 64.8 | 73.9 |  | 11.4 | 34.6 |
| MWCNT/Glu@RML | 0.0048 | 0.0128 |  | 144.6 | 54.6 |  | 25.3 | 25.5 |
| MWCNT/Gly@RML | 0.0063 | 0.0073 |  | 110.4 | 94.8 |  | 19.3 | 44.4 |

**Table S3.** Kinetic parameters of free and immobilized RML derivatives towards *p*-NPP. The studies were performed in optimal pH and temperature of each RML sample. Data are presented as mean ± SD (n = 3).

| **Enzymes** | ***Vmax***  **(U/mg prot)** | ***K_m_* [mM]** | ***k_cat_* (min^-1^)** | **Catalytic efficiency**  ***k_cat_*/*K_m_* (mM^-1^ min^-1^)** | **CER** |
| --- | --- | --- | --- | --- | --- |
| Free RML | 0.82 ± 0.22 | 3.14±0.2 | 43.4 | 13.8 | - |
| MWCNT/Gen@RML | 0.35 ± 0.15 | 2.94±0.1 | 27.6 | 9.38 | 0.7 |
| MWCNT/Glu@RML | 1.44 ± 0.21 | 3.26±0.2 | 62.7 | 19.2 | 1.4 |
| MWCNT/Gly@RML | 0.76 ± 0.15 | 3.42±0.3 | 38.4 | 11.2 | 0.8 |
